# FABP4/5-Mediated Lipid Reprogramming Is Associated with Immunosuppressive T Cell Crosstalk in Cervical Cancer After Chemoradiotherapy

**DOI:** 10.3390/biomedicines14092072

**Published:** 2026-09-15

**Authors:** Tianhan Xu, Mingjun Ma, Jiawen Zhang, Yanan Wang, Xiaoxia Tang, Sufang Wu

**Affiliations:** 1 Department of Obstetrics and Gynecology, Shanghai General Hospital, Shanghai Jiao Tong University School of Medicine, Shanghai 200080, China; 2 Department of Obstetrics and Gynecology, Tongji Hospital, School of Medicine, Tongji University, Shanghai 200065, China; 3 Tongji University Cancer Center, Shanghai Tenth People’s Hospital, Tongji University School of Medicine, Shanghai 200072, China; 4 The Affiliated Taizhou People’s Hospital of Nanjing Medical University, Taizhou 225300, China

**Keywords:** cervical cancer, concurrent chemoradiotherapy (CCRT), FABP4/5, lipid metabolism reprogramming, T cell immune suppression, single-cell transcriptome, tumor microenvironment

## Abstract

**Background:** Concurrent chemoradiotherapy (CCRT) is the standard treatment for locally advanced cervical cancer, but resistance and recurrence remain major clinical challenges. Tumor immune–metabolic reprogramming profoundly affects treatment response and immune evasion; however, the molecular mechanisms of lipid metabolism remodeling and its interaction with T cells after CCRT in cervical cancer are still unclear. This study aimed to investigate how CCRT-induced metabolic changes in cervical cancer cells influence T cell function and immune evasion. **Methods:** We performed metabolic pathway activity analysis, cell–cell communication prediction, and transcriptomic analysis on paired single-cell transcriptomic data from three cervical cancer patients before and after CCRT (*n* = 3 paired). Validation was conducted using multiple independent GEO cohorts, in vitro cell culture experiments, and multi-sample immunofluorescence staining. **Results:** CCRT was associated with upregulation of *FABP4/5* in malignant epithelial cells and activation of the PPAR and adipocytokine signaling pathways, leading to lipid metabolic reprogramming. Epithelial cells with high *FABP4/5* expression showed enhanced predicted intercellular communication with T cells via the computationally inferred ligand–receptor axes *PTGER4* and *CXCR4*. T cells with high communication intensity exhibited an immunosuppressive phenotype signature, accompanied by metabolic alterations in lipid and carbohydrate pathways and enrichment of regulatory T cell subsets. Validation experiments confirmed the association between *FABP4/5* and inhibitory T cells. **Conclusions:** This study suggests that *FABP4/5*-mediated lipid metabolism reprogramming may represent a key mechanism contributing to the immunosuppressive crosstalk between cervical cancer epithelial cells and T cells after CCRT. Targeting this axis may reverse T cell dysfunction and provide a potential immunometabolic strategy to reduce post-CCRT recurrence.

## 1. Introduction

Cervical cancer is the fourth most common malignancy in women worldwide, with approximately 660,000 new cases and 340,000 deaths annually, posing a serious threat to women’s health [1]. For locally advanced cervical cancer (FIGO stage IB3–IVA), concurrent chemoradiotherapy (CCRT) is the standard treatment that significantly improves local control and survival [2]. However, about 30% of patients develop resistance or recurrence, leading to treatment failure [3], underscoring the urgent need to elucidate resistance mechanisms and identify new therapeutic targets.

Immune–metabolic reprogramming in the tumor microenvironment critically regulates treatment response and tumor recurrence [4]. Metabolic alterations not only fuel tumor proliferation and survival but also shape an immunosuppressive niche by modulating immune cell function [5]. Lipid metabolism—including fatty acid uptake, oxidation, and storage—has gained attention for enabling tumor cells to withstand chemoradiotherapy stress [6,7]. Studies show that CCRT induces lipid remodeling in tumor cells, promoting adaptation to oxidative stress and DNA damage while fostering an immunosuppressive microenvironment through metabolite-mediated intercellular communication, collectively contributing to resistance [8,9]. Nevertheless, the metabolic reprogramming of cervical cancer cells after CCRT and their interaction with immune cells remain unexplored at single-cell resolution, necessitating deeper investigation into heterogeneity and metabolic-immune dynamics.

Fatty acid-binding proteins (FABPs) are key intracellular lipid chaperones. Among them, *FABP4* and *FABP5* (*FABP4/5*) are aberrantly expressed in multiple cancers [10,11] and contribute to tumor progression and therapy tolerance by reshaping lipid metabolism in tumor and immune cells, suggesting their potential as therapeutic targets in immuno-oncology [12,13,14]. Although *FABP5* overexpression in cervical cancer has been linked to lymph node metastasis [15,16], its expression dynamics, regulatory mechanisms, and relationship with resistance to concurrent chemoradiotherapy (CCRT) remain largely unexplored, representing a critical gap in understanding therapeutic resistance in this context.

Metabolic competition between tumor and immune cells is pivotal for immune evasion [17,18]. Tumor cells outcompete T cells for glucose and lipids, suppressing effector functions [19], while tumor-derived metabolites skew T cells toward immunosuppressive phenotypes [20,21]. Lipid metabolism plays dual roles: supporting tumor survival [22,23] while dictating T cell differentiation fate [24]. Thus, deciphering the metabolic dialog between tumor cells and T cells is crucial for understanding immune evasion and treatment resistance.

Currently, research on dynamic metabolic and immune changes after CCRT remains limited, particularly at single-cell resolution. Traditional bulk analyses fail to capture cellular heterogeneity and functional alterations in specific subpopulations. To address this, we utilized public single-cell transcriptomic data from cervical cancer patients pre- and post-CCRT to construct a high-resolution metabolic and immune landscape. We systematically analyzed treatment-associated alterations, focusing on *FABP4/5*-mediated lipid reprogramming and its role in regulating intercellular communication between cancer epithelial cells and T cells, leading to T cell functional remodeling. The above findings were further verified by cell experiments and multiplex immunofluorescence of tissues from 101 patients with cervical cancer. These results may provide new molecular targets for overcoming drug resistance and reducing recurrence of concurrent chemoradiotherapy in cervical cancer.

## 2. Materials and Methods

### 2.1. Cell Culture

Human cervical cancer cell lines HeLa and SiHa were obtained from the American Type Culture Collection (ATCC, Manassas, VA, USA). HeLa cells were cultured in Dulbecco’s modified Eagle’s medium (DMEM) supplemented with 10% fetal bovine serum (FBS) and 1% penicillin-streptomycin. SiHa cells were maintained in minimum essential medium (MEM) containing 10% FBS and 1% penicillin-streptomycin. All cells were incubated at 37 °C in a humidified atmosphere with 5% CO_2_.

### 2.2. RT-qPCR

Total RNA was extracted from HeLa and SiHa cells using TRIzol reagent (Invitrogen, Carlsbad, CA, USA) according to the manufacturer’s protocol. Reverse transcription was performed using a HiScript III RT SuperMix for qPCR (Vazyme, Nanjing, China) to synthesize cDNA. Quantitative real-time PCR was carried out with ChamQ Universal SYBR qPCR Master Mix (Vazyme, Nanjing, China) on a CFX96 Real-Time PCR System (Bio-Rad, Hercules, CA, USA). The primer sequences used were as follows: *GAPDH* (forward: ACAACTTTGGTATCGTGGAAGG, reverse: GCCATCACGCCACAGTTTC); *HOXB2* (forward: CGCCAGGATTCACCTTTCCTT, reverse: CCCTGTAGGCTAGGGGAGAG); *EHF* (forward: CAGTGCAGTAGTGACCTGTTC, reverse: CTGTGCTACCATAGTTGGTGTC); *ELF3* (forward: GGCCGATGACTTGGTACTGAC, reverse: GCTTGCGTCGTACTTGTTCTTC).

### 2.3. Multiplex Immunofluorescence

Cervical cancer tissue sections were deparaffinized, rehydrated, and subjected to antigen retrieval using citrate buffer (pH 6.0). After blocking with 5% normal goat serum, sections were incubated overnight at 4 °C with primary antibodies from Proteintech (Rosemont, IL, USA): anti-FABP4 (12802-1-AP), anti-FABP5 (12348-1-AP), and anti-CD25/IL-2RA (83896-1-RR). Following washing, sections were incubated with appropriate fluorophore-conjugated secondary antibodies. Nuclei were counterstained with DAPI. Images were acquired using a fluorescence microscope. This study was approved by the Ethics Committee (approval No. 2025KS731).

### 2.4. Data Source

The single-cell transcriptomic data used in this study originated from the public gene expression database (Gene Expression Omnibus, GEO) dataset GSE236738, which included tumor tissues from 3 pairs of cervical cancer patients before concurrent chemoradiotherapy (CCRT) and their paired samples after CCRT. Additional transcriptomic datasets, including GSE9750, GSE52903 and GSE39001, were obtained from GEO for validation analyses. The transcriptomic data for survival analysis and clinical information were sourced from the Cancer Genome Atlas (TCGA) cervical cancer cohort (TCGA-CESC). The relevant charts were generated based on online analysis on the GEPIA website (http://gepia.cancer-pku.cn/) (accessed on 15 May 2026).

### 2.5. Single-Cell Data Processing

The Seurat package (version 4.3.0) was used to perform quality control and data normalization on the original single-cell expression matrix. The filtering criteria included: (1) between 200 and 6000 genes detected by each cell and (2) a percentage of mitochondrial genes of less than 20%. All six samples were then merged into a single cohort and jointly normalized using SCTransform, which corrects for sequencing-depth differences across samples. Dimensionality reduction was performed through principal component analysis (PCA), and graph-based clustering was performed on the first 20 principal components for cell grouping, with UMAP used for visualization. Cell types were annotated based on canonical marker genes in the merged cohort.

### 2.6. Metabolic Pathway Activity Score

Metabolic gene sets were obtained from the KEGG database. The activity scores of individual cells for specific metabolic pathways were calculated using the AUCell algorithm and validated using GSVA. Metabolic pathway activity was normalized by z-score and presented as heatmaps, bubble plots, and box plots. Differences in metabolic pathway activity before and after CCRT and among different cell subpopulations were evaluated using the Wilcoxon rank-sum test.

### 2.7. Differential Expression and Functional Enrichment

The FindMarkers function in Seurat, using the Wilcoxon rank-sum test, was used to identify differentially expressed genes (DEGs) between cell subpopulations or treatment groups. Genes with |log2FC| > 0.25 and an adjusted *p* value < 0.05 were considered differentially expressed. Gene Ontology (GO) and Kyoto Encyclopedia of Genes and Genomes (KEGG) enrichment analyses were performed using the clusterProfiler package (version 4.4.0). Gene set enrichment analysis (GSEA) was performed using the fgsea package (version 1.22.0) based on a pre-ranked gene list ordered by log2FC.

### 2.8. Spatiotemporal Analysis

The Slingshot and Monocle3 algorithms were used to construct a pseudotime trajectory of cervical cancer epithelial cells and characterize epithelial differentiation and the dynamic expression patterns of FABP4/5 along the trajectory. Changes in FABP4/5 expression were analyzed in relation to epithelial differentiation. The analysis was performed using the Slingshot (version 2.8.0) and Monocle3 (version 1.3.1) packages in R (version 4.2.1).

### 2.9. Transcription Factor Regulatory Network

The SCENIC algorithm (pySCENIC 0.12.1) was used to infer transcription factor (TF) activity at the single-cell level. Based on the human TF database and motif information, AUCell was used to quantify the activity of each TF regulatory module. TFs showing differential activity between FABP4/5-high and FABP4/5-low epithelial cells were identified, and their target gene sets were used to construct the upstream regulatory networks of FABP4 and FABP5. Target gene counts were further analyzed to visualize the regulatory enrichment of core TFs in the high-expression subgroups.

### 2.10. Intercellular Communication Analysis

Epithelial cells were stratified into *FABP4*-high/low and *FABP5*-high/low subgroups using the median expression value across all epithelial cells in the merged cohort (a global median split of log-normalized expression). Specifically, because 85.2% of epithelial cells showed no detectable *FABP4* transcript, the global median for *FABP4* was 0, and the split was effectively equivalent to detection-positive versus detection-negative cells (*FABP4*-high, *n* = 432; *FABP4*-low, *n* = 2487); for *FABP5*, the median value was 0.693 (log-normalized expression), yielding *FABP5*-high, *n* = 1161, and *FABP5*-low, *n* = 1758. CellChat (version 1.6.0) was used to infer intercellular communication based on the CellChatDB l– database. Communication probabilities were estimated from the average expression of ligands and receptors in each cell subgroup and evaluated using permutation tests. FABP4-high and FABP5-high epithelial cells were defined as signal-sending populations, with other cell types considered potential signal-receiving populations. Communication networks were visualized using bubble plots, network diagrams, heatmaps, and circular plots. Key l– pairs and signaling pathways were identified based on communication probability and signal contribution analysis.

NicheNet (version 1.1.0) was subsequently used to investigate potential ligand–target regulatory relationships between FABP4/5-high epithelial cells and T cells. FABP4/5-high epithelial cells were defined as sender cells and T cells as receiver cells. Candidate ligands were ranked based on their predicted regulatory activity using l– and signaling databases, and their potential target genes were visualized using heatmaps and network diagrams. Key receptors were further identified by integrating l– predictions with communication strength scores.

### 2.11. Statistics

All statistical analyses and visualizations in this study were conducted using the R language (version 4.2.1). The Wilcoxon rank sum test was used for comparisons between the two groups, and the Kruskal–Wallis test was used for comparisons among multiple groups. The analysis of differentially expressed genes utilized the FindMarkers function from the Seurat package (version 4.1.1) (based on the Wilcoxon test), with a significance threshold set at |log_2_FC| > 0.25 and an adjusted *p* value < 0.05. The TCGA survival analysis was performed using the Kaplan–Meier method to draw survival curves, and the Log rank test was used to compare differences between groups.

## 3. Results

### 3.1. CCRT Remodels the Cellular Composition and Metabolic Landscape of the Cervical Cancer Microenvironment

**Figure 1:**
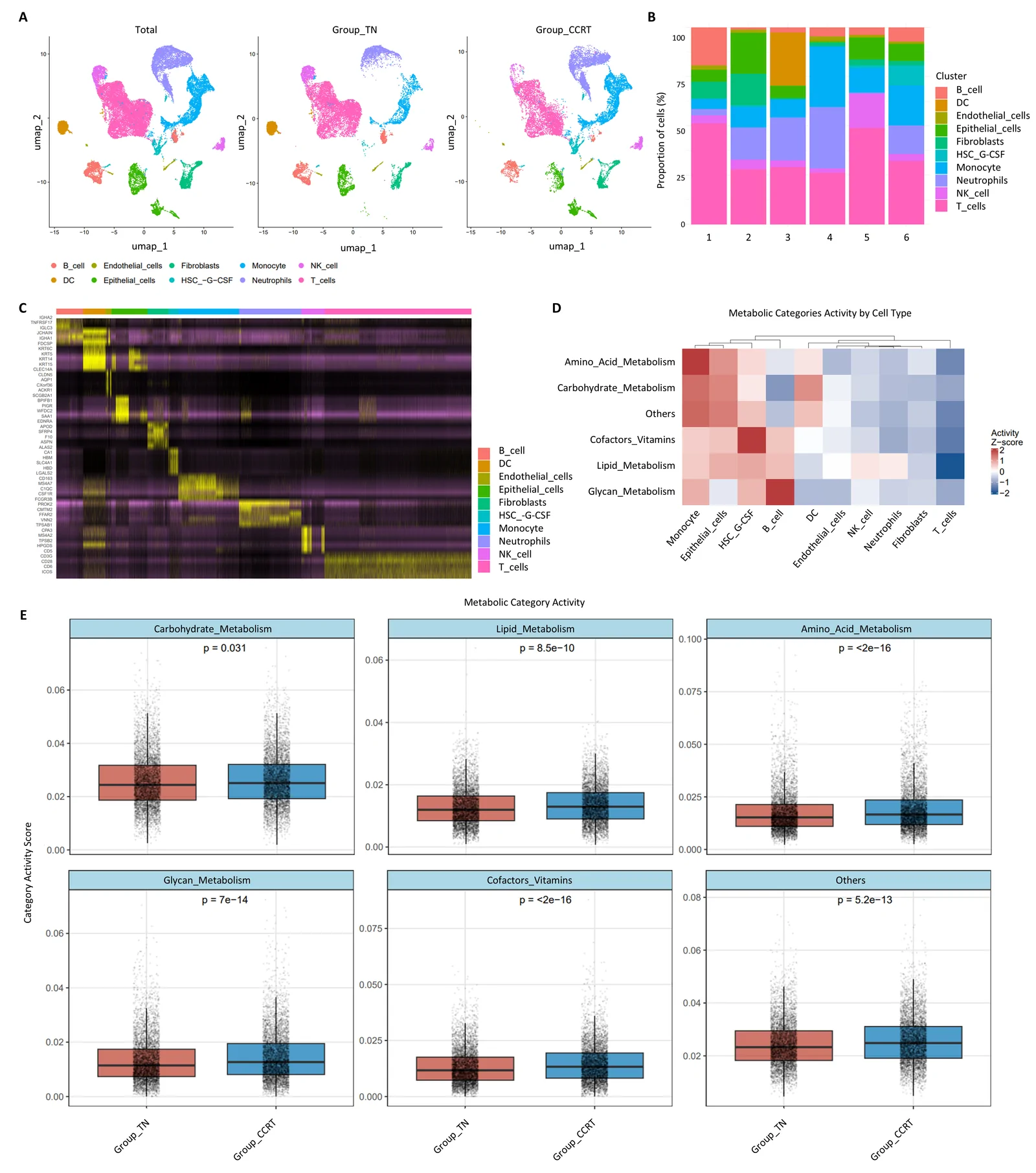
Single-cell transcriptomic and metabolic landscape of cervical cancer before and after CCRT. (**A**) UMAP visualization of major cell types in cervical cancer, including the overall cohort and subgroups before (Group_TN) and after (Group_CCRT) treatment. (**B**) Bar plots showing the proportional distribution of different cell types across individual patient samples. (**C**) Heatmap depicting marker gene expression for each cell type, with hierarchical clustering defining cell identity. (**D**) Heatmap of metabolic pathway activity z-scores stratified by major cell types. (**E**) Box plots comparing major metabolic pathway activity scores between pre-CCRT (Group_TN) and post-CCRT (Group_CCRT) samples, with *p*-values indicating statistical significance.

To investigate the impact of concurrent chemoradiotherapy (CCRT) on the cervical cancer tumor microenvironment (TME), we analyzed paired single-cell transcriptomic data from three patients before (Group_TN) and after (Group_CCRT) treatment. After quality control, a total of 33,742 cells were retained (16,926 pre-CCRT [Group_TN] and 16,816 post-CCRT [Group_CCRT]) from the six samples of three paired patients, and 10 major cell types were annotated, including B cells, dendritic cells (DCs), endothelial cells, epithelial cells, fibroblasts, hematopoietic stem cells (HSC-G-CSF), monocytes, neutrophils, natural killer cells (NK cells), and T cells (Figure 1A, Supplementary Figure S1A,B). Cell composition analysis revealed substantial remodeling of the TME following CCRT (Figure 1B), and marker gene expression confirmed the reliability of the clustering (Figure 1C, Supplementary Figure S1C).

To assess CCRT-induced metabolic reprogramming, we employed the AUCell method with KEGG pathways to score metabolic activities across 33,742 cells. Significant metabolic heterogeneity was observed among cell populations (Figure 1D, Supplementary Figure S1D). Notably, metabolic pathway activities broadly increased after treatment, indicating widespread metabolic remodeling induced by CCRT (Figure 1E).

### 3.2. The Specific Transcriptome of Cervical Cancer Epithelial Cells After CCRT Is Enriched in Lipid Metabolism Pathways

**Figure 2:**
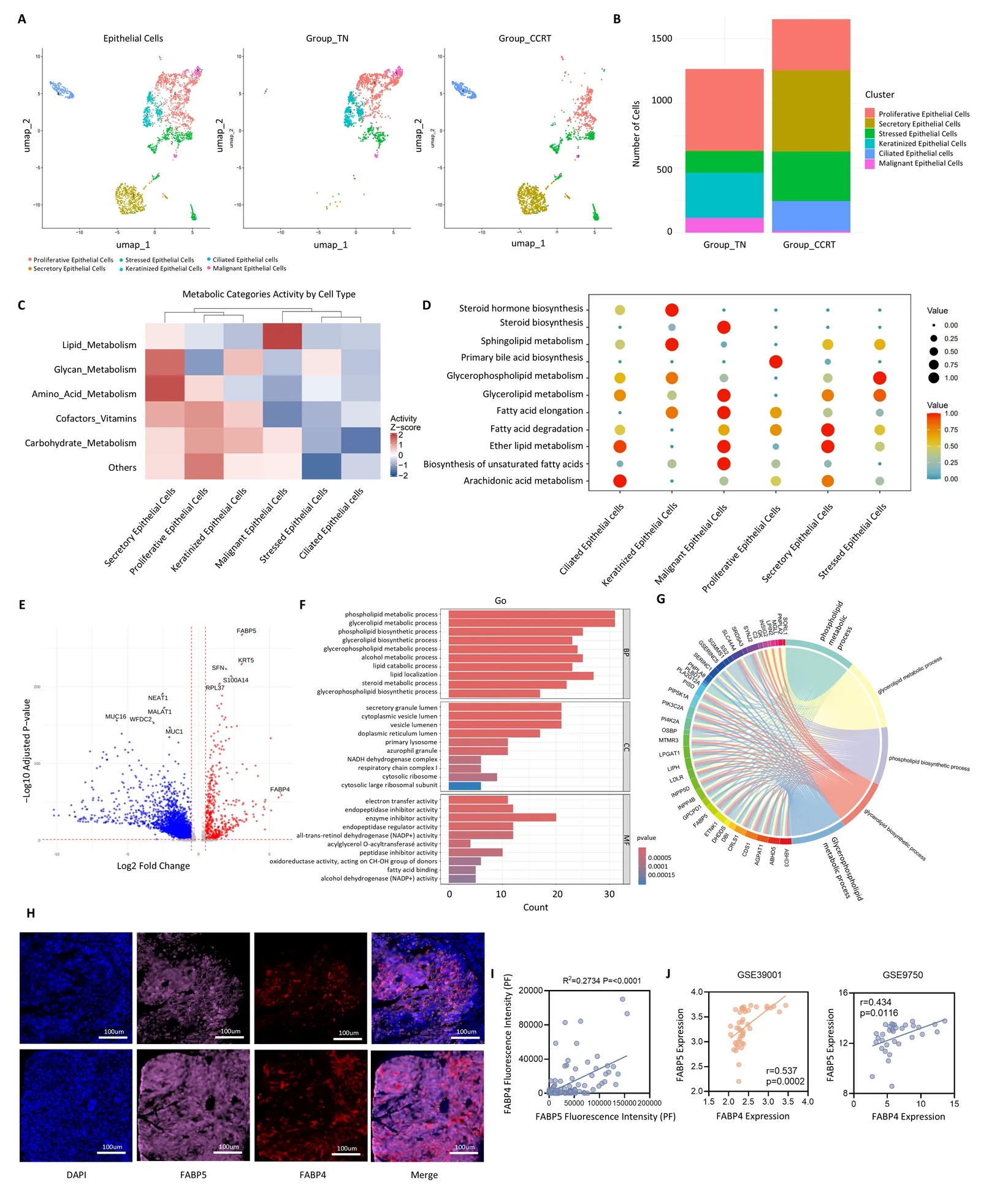
Single-cell transcriptomic and metabolic landscape of cervical cancer epithelial cells before and after CCRT. (**A**) UMAP visualization of epithelial cell clusters in the overall cohort and subgroups before (Group_TN) and after (Group_CCRT) treatment. (**B**) Bar plots showing the proportional distribution of epithelial cell clusters in Group_TN and Group_CCRT samples. (**C**) Heatmap of metabolic pathway activity z-scores stratified by major epithelial cell types. (**D**) Bubble plot of lipid metabolism pathway activity scores stratified by major epithelial cell types. (**E**) Volcano plot of differentially expressed genes between malignant epithelial cells and other epithelial cell types. (**F**) Bar plot of GO enrichment analysis for identified differentially expressed genes. (**G**) Chord diagram of GO enrichment analysis illustrating functional relationships among differentially expressed genes. (**H**) Representative immunofluorescence images of FABP4 and FABP5 staining. (**I**) Pearson correlation analysis was used to evaluate the correlation between FABP4 and FABP5 immunofluorescence expression intensity in 101 cervical cancer tissues. (**J**) Pearson correlation analysis was used to evaluate the correlation between FABP4 and FABP5 gene expression levels in the GSE39001/GSE9750 datasets.

As the core target cells of cervical cancer, epithelial cells were further analyzed to delineate CCRT-specific transcriptional alterations. Re-clustering of 2919 epithelial cells from Group_TN and Group_CCRT samples identified six distinct subpopulations (Figure 2A, Supplementary Figure S2A). Their distribution patterns shifted markedly after CCRT, with proliferative and malignant epithelial cells showing significant proportion decreases, indicating effective treatment response and selective remodeling of epithelial composition (Figure 2B).

Metabolic heterogeneity among epithelial subpopulations was assessed using AUCell scoring of KEGG pathways in 2919 epithelial cells. Malignant epithelial cells exhibited significantly elevated lipid metabolism pathway activity compared with other subtypes, including steroid biosynthesis, fatty acid elongation, and biosynthesis of unsaturated fatty acids (Figure 2C,D), and were strongly associated with post-CCRT lipid metabolic reprogramming.

Differential expression analysis revealed pronounced transcriptional remodeling in malignant epithelial cells, with *FABP5* as the most significantly upregulated gene, and concurrent *FABP4* upregulation (Figure 2E). GO and KEGG enrichment analyses confirmed that upregulated genes were strongly associated with lipid metabolism processes—fatty acid metabolism, lipid transport, fatty acid binding, and fatty acid beta-oxidation (Figure 2F, Supplementary Figure S2B,C). The chord diagram further illustrated gene–function relationships within these metabolic modules (Figure 2G).

Pearson correlation analysis of immunofluorescence staining of FABP4 and FABP5 in 101 cervical cancer tissues showed a significant positive correlation between their expression intensity (Figure 2H,I). Analysis of transcriptome data from GSE39001 and GSE9750 further supported this association (Figure 2J). Taken together, these results establish lipid metabolic reprogramming as a central feature of the response of malignant epithelial cells to concurrent chemoradiotherapy and highlight the importance of FABP4/5 as a key candidate for mechanistic studies.

### 3.3. FABP4 and FABP5 Are Highly Expressed in Epithelial Cells After Treatment and Are Related to Cell Differentiation

**Figure 3:**
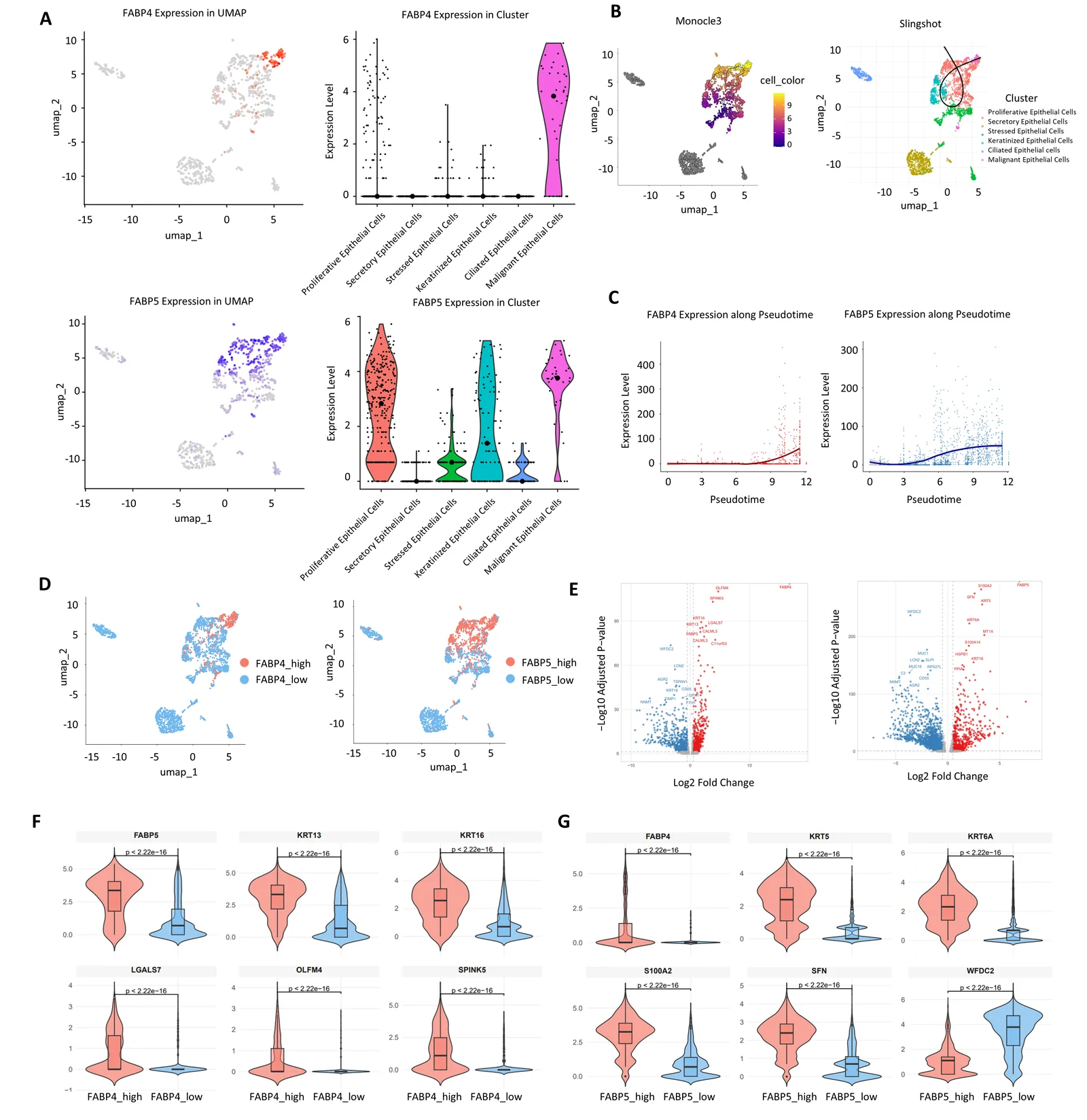
Expression and functional characteristics of *FABP4* and *FABP5* in cervical cancer epithelial cells. (**A**) UMAP visualization and violin plots showing *FABP4* and *FABP5* expression across distinct epithelial cell clusters. (**B**) Pseudotime trajectory analysis of epithelial cell differentiation constructed using Slingshot and Monocle3 algorithms. (**C**) Dynamic changes in *FABP4* and *FABP5* expression along the pseudotime trajectory. (**D**) UMAP visualization of epithelial cells stratified by *FABP4* (left) and *FABP5* (right) expression levels (high vs. low). (**E**) Volcano plots of differentially expressed genes (DEGs) between *FABP4/5*-high and *FABP4/5*-low epithelial cell subpopulations. (**F**) Violin plots showing representative differentially expressed genes between *FABP4*-high and *FABP4*-low epithelial cell subpopulations. (**G**) Violin plots showing representative differentially expressed genes between *FABP5*-high and *FABP5*-low epithelial cell subpopulations.

Given the core role of lipid metabolism reprogramming and significant *FABP4/5* upregulation in malignant epithelial cells, we further characterized their expression patterns and functional relevance. UMAP and violin plots confirmed that *FABP4* and *FABP5* were predominantly expressed in malignant and proliferative epithelial cells (Figure 3A). Pseudotime trajectory analysis (Slingshot and Monocle3) revealed progressively increasing *FABP4/5* expression along the epithelial differentiation trajectory (Figure 3B,C, Supplementary Figure S3A,B), suggesting their involvement in malignant differentiation.

Epithelial cells were stratified into *FABP4*-high/low and *FABP5*-high/low subgroups using the median expression value across all epithelial cells in the merged cohort (*FABP4*-high, *n* = 432; *FABP4*-low, *n* = 2487; *FABP5*-high, *n* = 1161, and *FABP5*-low, *n* = 1758, Figure 3D). Notably, the high-expression subgroup exhibited marked upregulation of KRT family members (*KRT5*, *KRT6A*, *KRT13*, *KRT16*)—key markers of cervical squamous epithelial malignant transformation and abnormal differentiation—along with proliferation- and stemness-associated genes, including *OLFM4*, *SFN*, and *LGALS7* (Figure 3E–G, Supplementary Figure S3C,D). These findings suggest that high FABP4/5 expression is associated with lipid metabolic reprogramming and malignant epithelial phenotypes, particularly through changes in proliferation- and differentiation-associated genes.

### 3.4. High FABP4/5 Expression Is Associated with PPAR Signaling and Specific Transcription Factor Regulatory Networks in Epithelial Cells

To elucidate downstream pathways and upstream regulatory mechanisms of *FABP4/5*-mediated lipid metabolism reprogramming, we analyzed pathway activities and transcription factor (TF) networks in *FABP4/5*-high versus -low epithelial subgroups.

**Figure 4:**
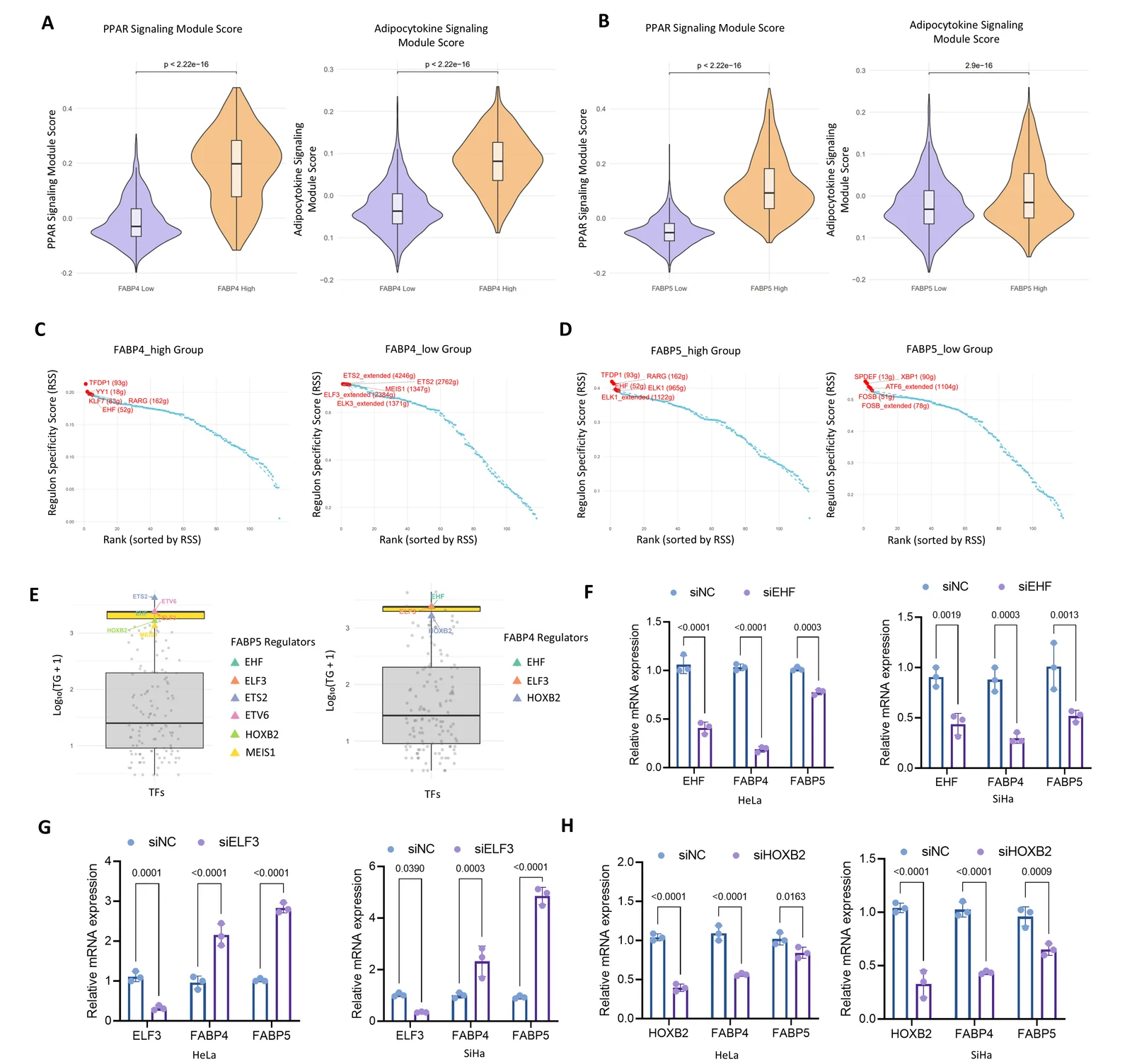
PPAR signaling pathway, adipocytokine pathway, and transcription factor regulatory networks in *FABP4/5*-expressing cervical cancer epithelial cells. (**A**,**B**) Violin plots comparing module scores of PPAR signaling and adipocytokine signaling pathways between epithelial subpopulations with high versus low *FABP4/5* expression. (**C**) Transcription factor (TF) activity analysis (SCENIC) showing TFs with differential activity between high-*FABP4* and low-*FABP4* epithelial subpopulations. (**D**) Transcription factor (TF) activity analysis (SCENIC) showing TFs with differential activity between high-*FABP5* and low-*FABP5* epithelial subpopulations. (**E**) Distribution and comparison of target gene counts of key transcription factors regulating *FABP4*. (**F**–**H**) RT-qPCR detection of *FABP4/5* mRNA after siRNA knockdown of *EHF* (**F**), *ELF3* (**G**), or *HOXB2* (**H**).

Compared with *FABP4*-low epithelial cells, *FABP4*-high epithelial cells showed significantly increased module scores of PPAR and adipocytokine signaling pathways (Figure 4A, Supplementary Figure S3E,F). Consistently, *FABP5*-high cells exhibited similar pathway enrichment (Figure 4B). These findings suggest a potential association between high *FABP4/5* expression and activation of PPAR signaling.

Gene set enrichment analysis (GSEA) of differentially expressed genes between high- and low-expression subgroups revealed that gene sets related to epithelial cell cycle, T cell cycle, and cervical cancer proliferation were significantly enriched in *FABP4/5*-high cells (Supplementary Figure S3G). This finding links lipid metabolism reprogramming to malignant proliferation and immune regulation, suggesting that *FABP4/5* may influence tumor cell cycle and T cell functional status.

Transcription factor activity analysis using SCENIC in 2919 epithelial cells revealed distinct regulatory networks between high- and low-expression subgroups. Key upstream TFs regulating *FABP4* included *EHF*, *ELF3*, and *HOXB2*, whereas *FABP5* was associated with *EHF*, *ELF3*, *ETS2*, *ETV6*, *HOXB2*, and *MEIS1*, all of which showed significant target gene enrichment in the high-expression subgroup (Figure 4C–E, Supplementary Figure S4A–D). Validation in HeLa and SiHa cells demonstrated that *EHF* and *HOXB2* positively regulated *FABP4/5*, while *ELF3* had the opposite effect (Figure 4F–H). These findings reveal a specific upstream transcriptional network regulating *FABP4/5* expression, providing potential regulatory nodes for targeted intervention.

### 3.5. Epithelial Cells with High Expression of FABP4/5 Have Enhanced Communication with T Cells

**Figure 5:**
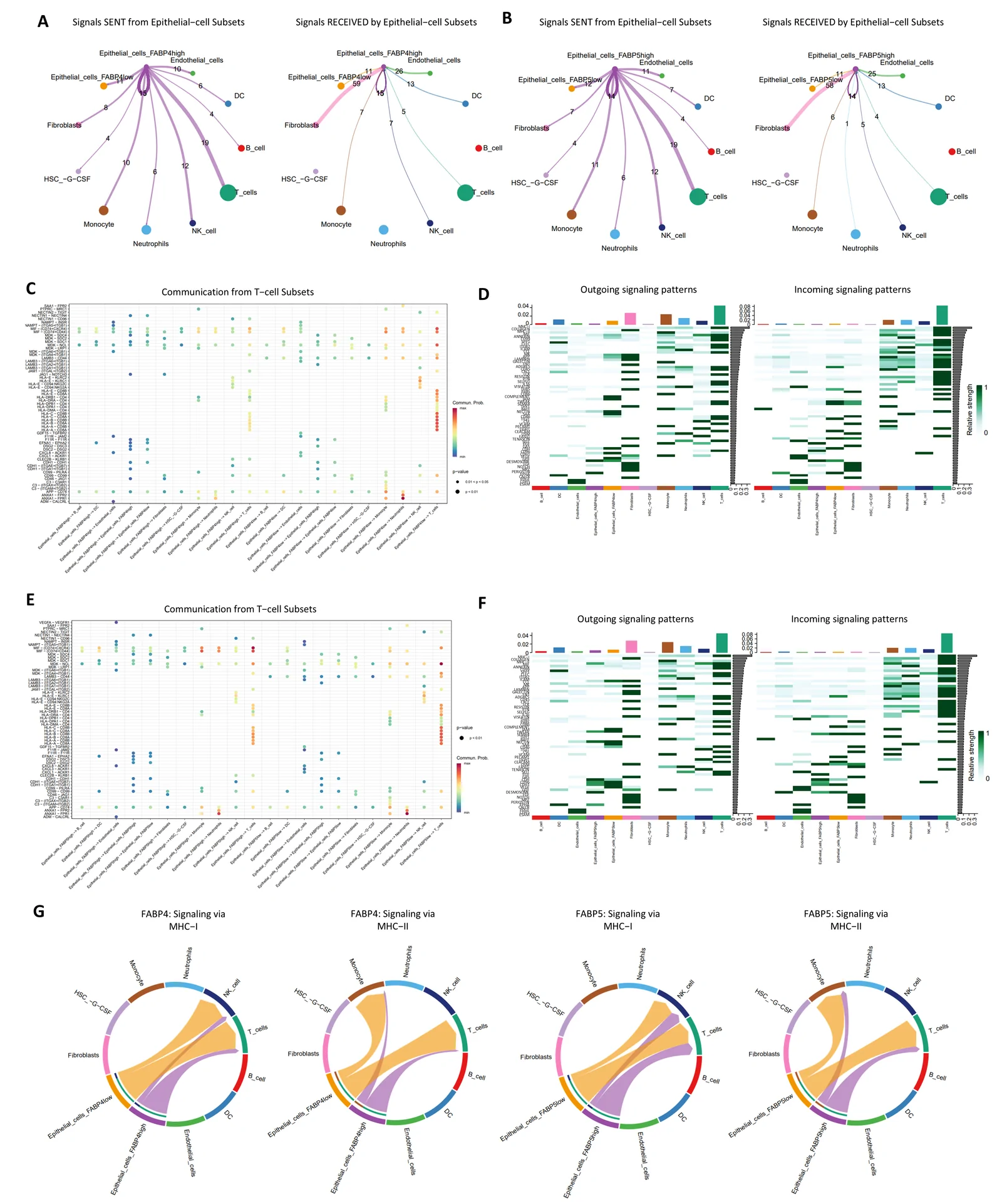
CellChat analysis of intercellular communication between *FABP4/5*-high/low epithelial cells and T cells. (**A**) Communication analysis between *FABP4*-high epithelial cells and other cell types. Left panel: outgoing signaling from *FABP4*-high epithelial cells; right panel: incoming signaling to *FABP4*-high epithelial cells. (**B**) Communication analysis between *FABP5*-high epithelial cells and other cell types. Left panel: outgoing signaling from *FABP5*-high epithelial cells; right panel: incoming signaling to *FABP5*-high epithelial cells. (**C**) Ligand–receptor or signaling pathway-mediated cell communication networks based on interaction analysis between *FABP4*-high epithelial cells and other cell types. (**D**) Outgoing and incoming signaling contributions based on interaction analysis between *FABP4*-high epithelial cells and other cell types. (**E**) Ligand–receptor or signaling pathway-mediated cell communication networks based on interaction analysis between *FABP5*-high epithelial cells and other cell types. (**F**) Outgoing and incoming signaling contributions based on interaction analysis between *FABP5*-high epithelial cells and other cell types. (**G**) Cellular pathway visualization.

To investigate the predicted interactions between *FABP4/5*-high epithelial cells and the immune microenvironment, we used CellChat to analyze intercellular communication, focusing on interactions with T cells. Compared with *FABP4*-low cells (*n* = 2487), *FABP4*-high epithelial cells (*n* = 432) showed stronger predicted communication with multiple immune cell populations, particularly T cells, and displayed prominent outgoing and incoming signaling patterns (Figure 5A). Consistently, *FABP5*-high epithelial cells (*n* = 1161) exhibited similar enhanced communication with T cells and other immune populations (Figure 5B), positioning them as potential signaling hubs in the tumor microenvironment.

L– interaction analysis revealed that *FABP4*-high epithelial cells possessed a markedly more complex communication network than their low-expression counterparts, with T cell interactions being the most prominent (Figure 5C). Key ligand–receptor pairs mediating this bidirectional communication included *MIF*-*CD74*/*CD44*, *ICAM1*-*LFA*-1, and *CXCL12*-*CXCR4* (Figure 5D). *FABP5*-high epithelial cells displayed highly similar interaction patterns (Figure 5E,F). Circular visualization further highlighted that both *FABP4*- and *FABP5*-high epithelial cells primarily engaged with T cells through MHC-I and MHC-II antigen presentation pathways (Figure 5G, Supplementary Figure S4E,F), underscoring their potential role in tissue microenvironment immune regulation.

### 3.6. NicheNet Analysis Identifies Potential Ligand–Receptor Interactions Between FABP4/5-High Epithelial Cells and T Cell Subsets

**Figure 6:**
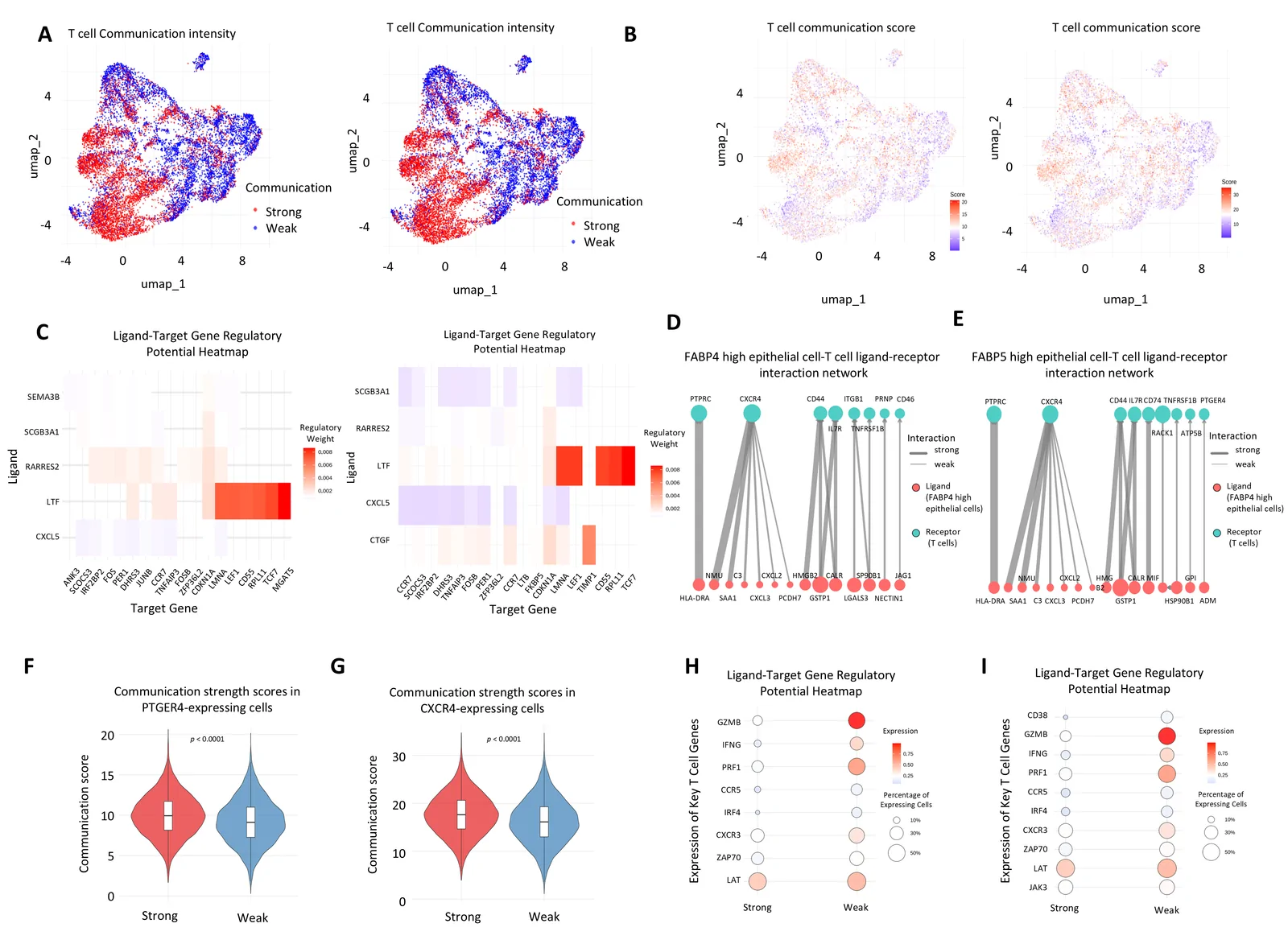
NicheNet analysis of T cell subpopulations and ligand–receptor regulatory networks mediated by *FABP4/5*-high epithelial cells. (**A**,**B**) UMAP visualization of T cells stratified by communication intensity (**A**) and colored by communication intensity scores (**B**) with *FABP4*-high (left) and *FABP5*-high (right) epithelial cells. (**C**) Heatmap showing regulatory potential of top-ranked active ligands and their top predicted target genes in *FABP4* (left) and *FABP5* (right) contexts. (**D**,**E**) Ligand–receptor interaction network diagrams mediated by *FABP4*-high (**D**) and *FABP5*-high (**E**) epithelial cells (17 ligands, 9 receptors). (**F**) Violin plot comparing communication intensity scores between strong and weak communication T cell subgroups expressing *PTGER4* (*FABP4* context, *p* < 0.0001). (**G**) Violin plot comparing communication intensity scores between strong- and weak-communication T cell subgroups expressing *CXCR4* (*FABP5* context, *p* < 0.0001). (**H**,**I**) Dot plots showing key T cell gene expression between strong- and weak-communication T cell subgroups in *FABP4* (**H**) and *FABP5* (**I**) contexts.

To delineate how *FABP4/5*-high epithelial cells modulate T cells, we employed NicheNet analysis. A total of 11,947 T cells were stratified into strong- and weak-communication subgroups based on communication intensity with *FABP4*-high (strong, *n* = 6940; weak, *n* = 5007) and *FABP5*-high (strong, *n* = 7031; weak, *n* = 4916) epithelial cells, respectively, with UMAP visualization revealing distinct clustering and intensity scores (Figure 6A,B). NicheNet analysis identified *SCGB3A1*, *RARRES2*, *LTF*, and *CXCL5* as the most active ligands in *FABP4/5*-expressing epithelial cells, predicted the target genes they may affect (Figure 6C), and constructed the corresponding ligand–receptor interaction network (Figure 6D,E, Supplementary Figure S5A–F). *PTGER4* emerged as a key receptor associated with *FABP4*-high epithelial cell communication, showing significantly higher intensity scores in the strong communication T cell subgroup (*p* < 0.0001) (Figure 6F, Supplementary Figure S5G–J). Similarly, *CXCR4* was identified as the core receptor for *FABP5*-high epithelial cell-mediated T cell regulation (Figure 6G).

Functional analysis revealed that T cells with strong communication to *FABP4/5*-high epithelial cells exhibited significantly lower expression of effector molecules *GZMB*, *IFNG*, and *PRF1* compared with weak communicators (Figure 6H,I), indicating an immunosuppressive state in these T cell subsets.

To confirm that the downstream results are robust, we re-derived the high/low stratification under four alternative cutoff schemes: (i) median of detection-positive cells only; (ii) global 75th percentile (top 25% vs the remainder); (iii) k-means clustering (k = 2) of log-expression; and (iv) per-sample median split. Under all five definitions (original + four alternatives), PPAR signaling scores were significantly higher in *FABP4/5*-high cells (10/10 comparisons, all high > low), and adipocytokine signaling scores were consistent in 9 of 10 comparisons. These results are presented in Supplementary Figure S6.

### 3.7. High-Communication-Intensity T Cells Exhibit Immunosuppressive Phenotypes and Are Accompanied by Metabolic Reprogramming Characteristics

Given the link between immune cell function and metabolic state, we characterized metabolic and transcriptional features of T cells with high communication intensity to *FABP5*-high epithelial cells (results for the *FABP4* context are shown in Supplementary Figures S7 and S8).

**Figure 7:**
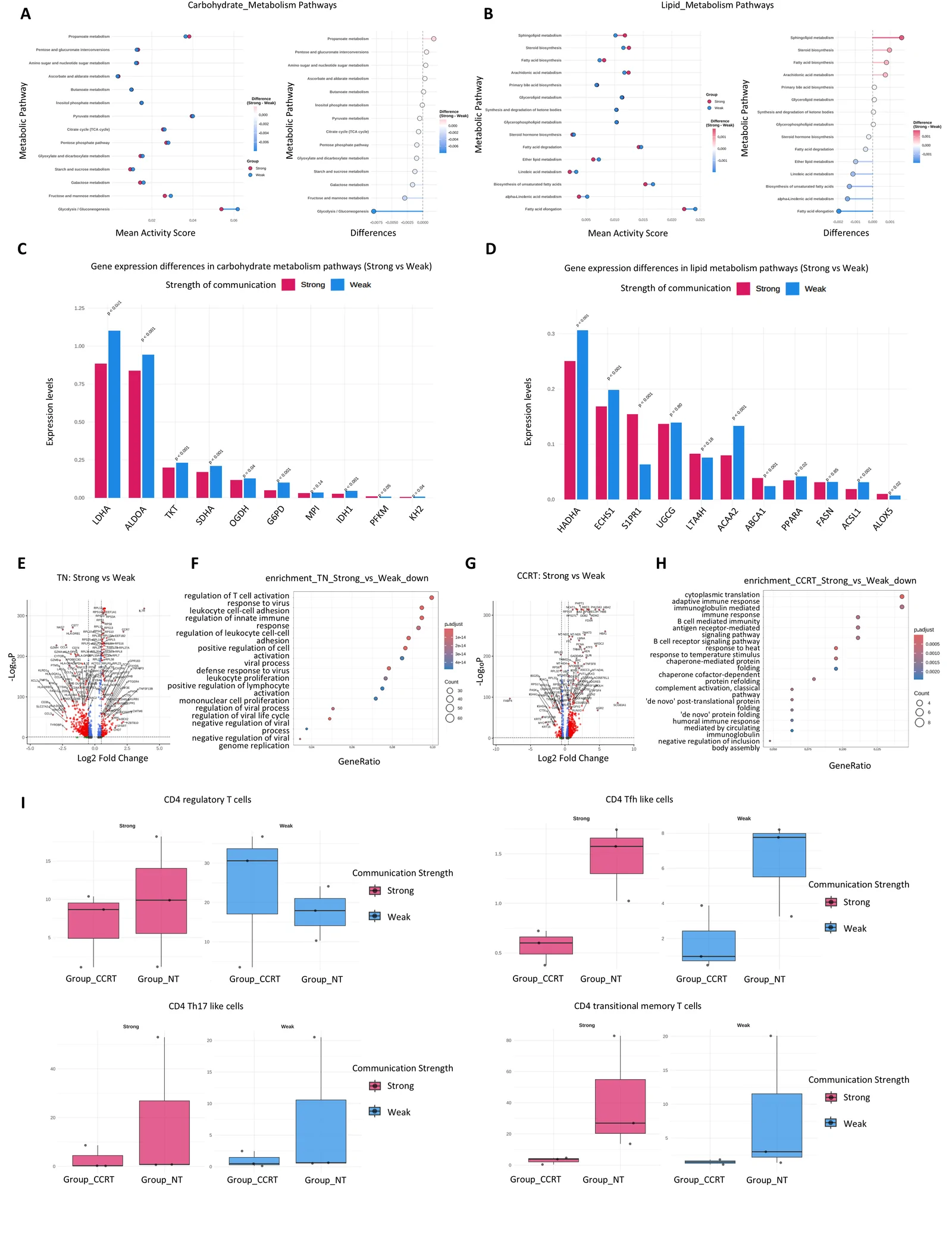
Metabolic reprogramming, transcriptomic alterations, and phenotypic heterogeneity of strong versus weak T cell subsets (FABP5 context) in cervical cancer. (**A**,**B**) Violin plots comparing lipid metabolism (**A**) and carbohydrate metabolism (**B**) pathway activity scores between strong- and weak-communication T cell subsets stratified by communication intensity with FABP5-high epithelial cells. (**C**,**D**) Bar plots showing expression differences in glycolysis and energy metabolism-related genes (**C**) and fatty acid metabolism-related genes (**D**) between strong- and weak-communication T cell subsets. (**E**,**F**) Volcano plot (**E**) and KEGG pathway enrichment analysis (**F**) of differentially expressed genes (DEGs) between strong- and weak-communication T cell subsets before treatment (TN). (**G**,**H**) Volcano plot (**G**) and KEGG pathway enrichment analysis (**H**) of differentially expressed genes (DEGs) between strong- and weak-communication T cell subsets after treatment (CCRT). (**I**) Proportional distribution of CD4+ regulatory T cells, CD4+ Tfh-like cells, CD4+ Th17-like cells, and CD4+ transitional memory T cells between strong- and weak-communication T cell subsets.

Metabolic pathway analysis revealed that high-communication T cells exhibited enhanced synthetic metabolism (propanoate metabolism, fatty acid biosynthesis) alongside reduced catabolic and oxidative stress pathways (fatty acid degradation, inositol phosphate metabolism, pentose phosphate pathway) compared with weak-communication T cells (Figure 7A,B), indicating a metabolic shift toward synthesis over degradation.

Consistent with this, high-communication T cells showed downregulation of glycolysis genes (*LDHA*, *ALDOA*, *HK2*), TCA cycle and oxidative phosphorylation genes (*SDHA*, *OGDH*, *IDH1*), and fatty acid degradation genes (*HADHA*, *ECHS1*, *ACAA2*), while lipid metabolism and synthesis genes (*S1PR1*, *ABCA1*, *ALOX5*) were upregulated (Figure 7C,D).

Transcriptional comparison between strong- and weak-communication subgroups revealed treatment-dependent functional remodeling. Before CCRT, downregulated genes in high-communication T cells were enriched in T cell activation pathways (Figure 7E,F). After CCRT, adaptive immune response and immunoglobulin-mediated immune response pathways were significantly suppressed (Figure 7G,H), suggesting that CCRT is associated with a shift in T cells from pre-CCRT activation inhibition to post-CCRT immune response modulation.

Analysis of T cell subtype composition showed that high-communication T cells contained significantly higher proportions of CD4+ regulatory T cells (Treg), CD4+ Tfh-like cells, and CD4+ Th17-like cells, with a corresponding decrease in CD4+ transitional memory T cells (Figure 7I). This preferential enrichment of immunosuppressive-associated subtypes suggests that T cells with high predicted communication intensity to *FABP5*-high epithelial cells display an immunosuppressive phenotype signature.

### 3.8. Correlation Analysis of FABP4/5 Expression and CD25^+^ Cell Infiltration with Clinicopathological Characteristics in Cervical Cancer Tissues

**Figure 8:**
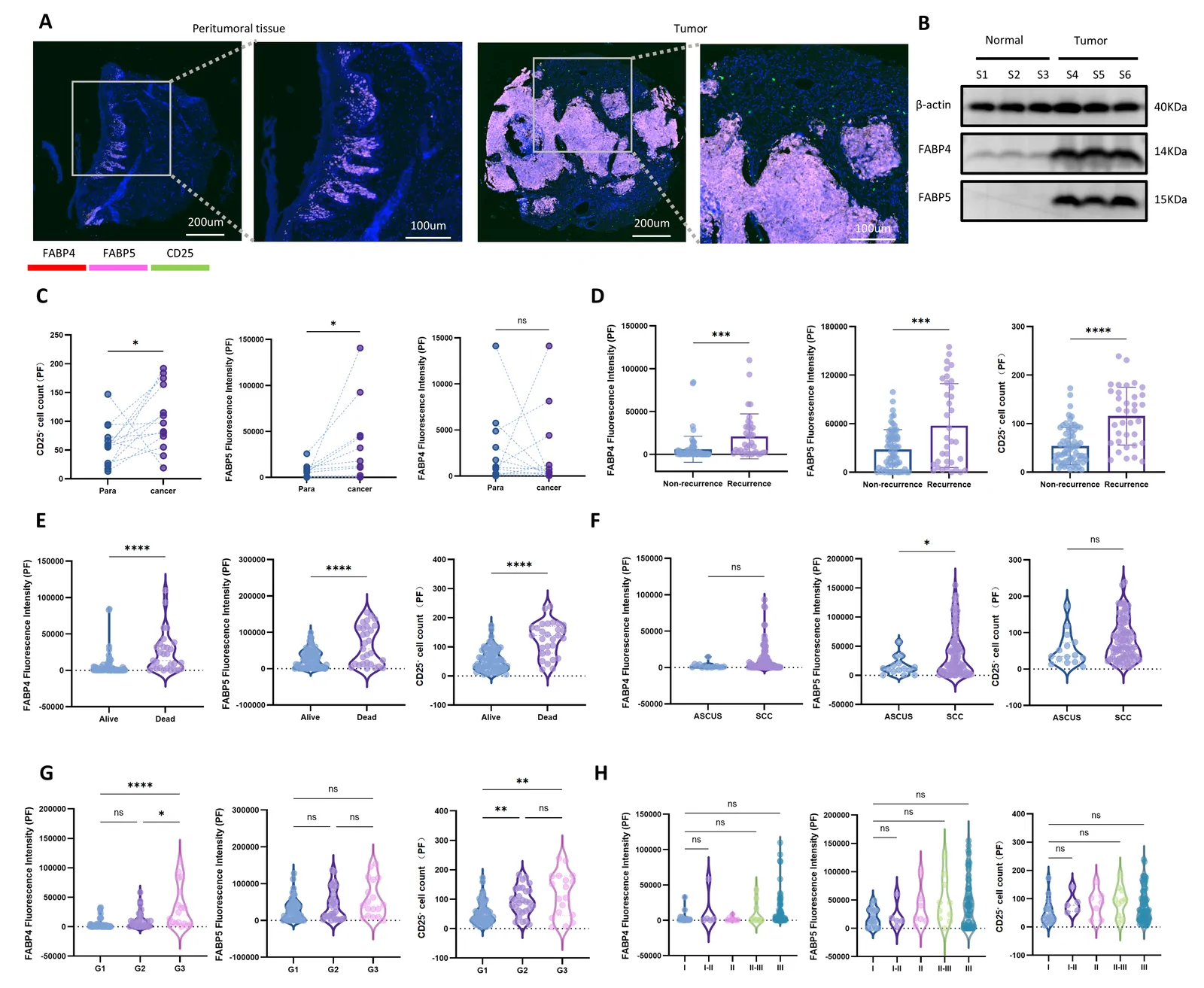
Clinicopathological significance of FABP4/5 expression and CD25^+^ cell infiltration in cervical cancer. (**A**) Representative immunofluorescence images of FABP4/5 expression in cervical cancer and adjacent non-cancerous tissues. (**B**) Western blot analysis of FABP4/5 protein expression in cervical cancer and adjacent non-cancerous tissues. (**C**) Paired *t*-test comparing FABP4/5 immunofluorescence intensity between cancer tissues and paired adjacent non-cancerous tissues (*n* = 13). (**D**) Independent *t*-test comparing FABP4/5 fluorescence intensity and CD25^+^ cell count between recurrence (*n* = 35) and non-recurrence (*n* = 66) groups. (**E**) Independent *t*-test comparing FABP4/5 fluorescence intensity and CD25^+^ cell count between deceased (*n* = 29) and alive (*n* = 72) groups. (**F**) Independent *t*-test comparing FABP4/5 fluorescence intensity and CD25^+^ cell count between ASCUS (*n* = 15) and SCC (*n* = 79) groups. (**G**) Kruskal–Wallis H test for differences in FABP4/5 fluorescence intensity and CD25^+^ cell count among groups: G1 (*n* = 55), G2 (*n* = 28), G3 (*n* = 18). (**H**) Kruskal–Wallis H test for differences in FABP4/5 fluorescence intensity and CD25^+^ cell count among histological grades: grade I (*n* = 16), grade I–II (*n* = 5), grade II (*n* = 7), grade II–III (*n* = 10), grade III (*n* = 63). * *p* < 0.05, ** *p* < 0.01, *** *p* < 0.001, **** *p* < 0.0001; ns, not significant.

We further analyzed the correlations of FABP4/5 expression and CD25^+^ cell infiltration with clinicopathological features in cervical cancer tissues. Immunofluorescence staining and Western blot results showed that FABP4/5 expression levels were elevated in cervical cancer tissues compared with adjacent non-cancerous tissues (Figure 8A,B). Paired *t*-tests confirmed that both FABP4/5 immunofluorescence intensity and CD25^+^ cell count were significantly higher in cancer tissues than in paired adjacent non-cancerous tissues (Figure 8C, paired samples [*n* = 13], *p* < 0.05; Supplementary Table S1). Regarding clinical outcomes, independent *t*-tests showed that the recurrence group (*n* = 35) had a higher FABP4/5 fluorescence intensity and CD25^+^ cell count than the non-recurrence group (*n* = 66); the deceased group (*n* = 29) also had higher values for the above indicators than the alive group (*n* = 72) (Figure 8D,E; Supplementary Tables S2 and S3). Comparison of different pathological types indicated that the SCC group (*n* = 79) had a higher FABP4/5 fluorescence intensity than the ASCUS group (*n* = 15) (Figure 8F; Supplementary Table S4). The GSE52903 dataset further confirmed that FABP5 gene expression in SCC was significantly higher than in ACC and ASCC (Supplementary Figure S9A; Supplementary Table S5). In addition, using the Kruskal–Wallis H test, significant differences in FABP4/5 fluorescence intensity and CD25^+^ cell count were found among clinical stage groups G3 (*n* = 18), G2 (*n* = 28), and G1 (*n* = 55), while among different histological grades (grade III, *n* = 63; grade II–III, *n* = 10; grade II, *n* = 7; grade I–II, *n* = 5; grade I, *n* = 16), although no statistical significance was reached, a certain trend was observed (Figure 8G,H; Supplementary Tables S6 and S7).

### 3.9. Correlation Between FABP4/5 Expression and CD25^+^ Cell Infiltration and Their Association with Patient Prognosis

**Figure 9:**
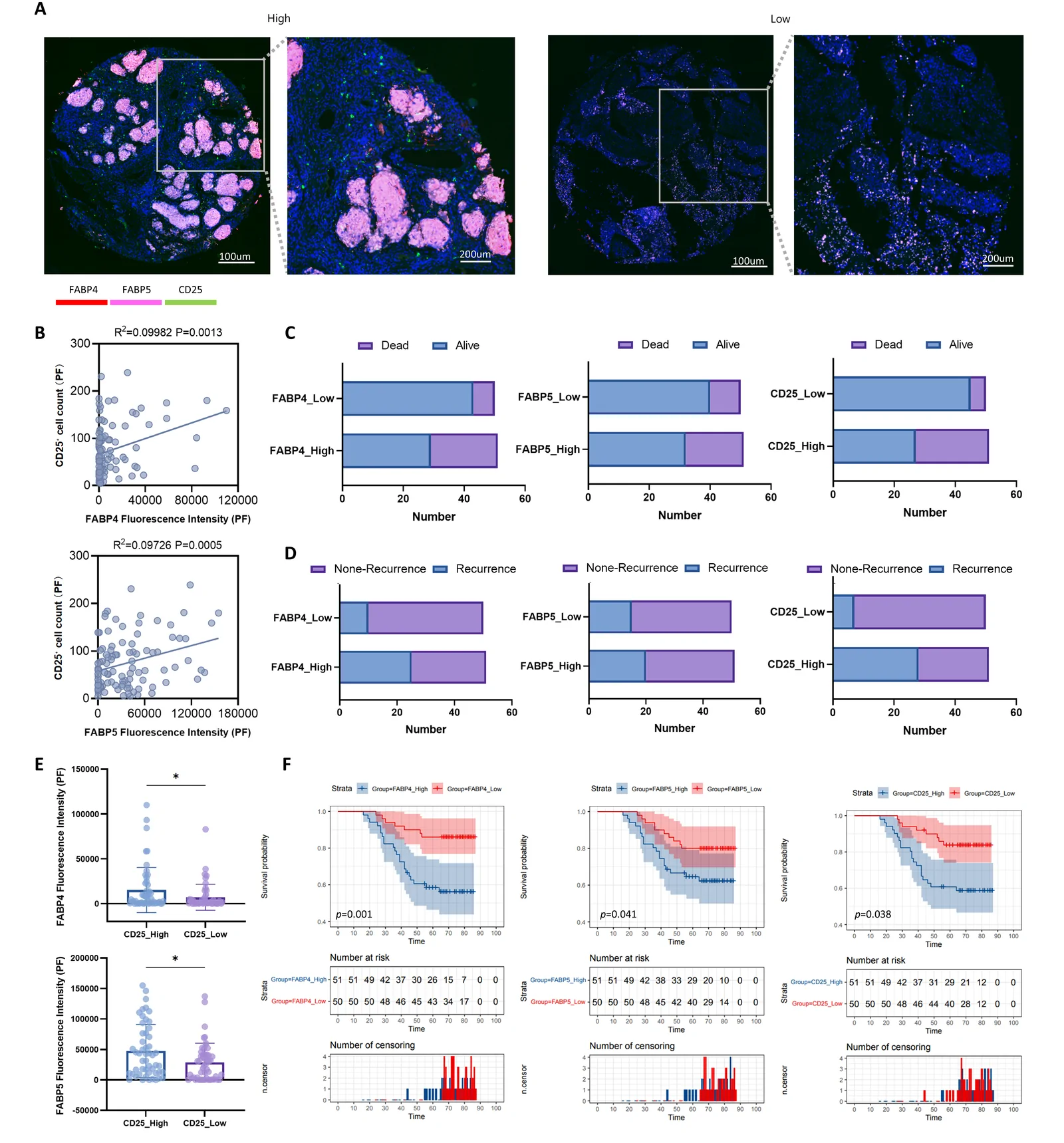
Correlation between FABP4/5 expression and CD25^+^ cell infiltration and their association with patient prognosis. (**A**) Representative immunofluorescence images of high and low FABP4/5 expression groups. (**B**) Correlation analysis between FABP4/5 fluorescence intensity and CD25^+^ cell infiltration level using Spearman rank correlation analysis. (**C**) Distribution of deceased and alive patients within groups defined by high/low FABP4/5 expression and high/low CD25^+^ cell infiltration levels. (**D**) Distribution of relapsed and non-relapsed patients within groups defined by high/low FABP4/5 expression and high/low CD25^+^ cell infiltration levels. (**E**) Comparison of FABP4/5 fluorescence intensity between groups with high (*n* = 51) and low (*n* = 50) CD25^+^ cell infiltration using independent *t*-test. (**F**) Survival curve analysis of subgroups stratified by high/low *FABP4/5* expression and high/low CD25^+^ cell infiltration levels using the Kaplan–Meier method. * *p* < 0.05.

To further clarify the relationship between FABP4/5 expression and CD25^+^ cell infiltration and its clinical significance, the 101 cervical cancer tissue samples were stratified into high and low FABP4/5 expression groups using the median immunofluorescence intensity as the global cutoff. We first observed obvious differences between the high and low FABP4/5 expression groups in immunofluorescence images (Figure 9A). Correlation analysis showed a significant positive correlation between FABP4/5 fluorescence intensity and CD25^+^ cell infiltration level (Spearman rank correlation analysis, Figure 9B, Supplementary Figure S9B). Further observation of the distribution of deceased and alive patients (Figure 9C), as well as relapsed and non-relapsed patients (Figure 9D), in each subgroup suggested that high FABP4/5 expression and high CD25^+^ infiltration were associated with poor prognosis. Independent *t*-test results indicated that the CD25^+^ cell infiltration level in the high FABP4/5 expression group (*n* = 51) was significantly higher than that in the low expression group (*n* = 50); the FABP4/5 fluorescence intensity in the high CD25^+^ cell infiltration group (*n* = 51) was also significantly higher than that in the low infiltration group (*n* = 50) (Figure 9E, Supplementary Figure S9C). Patients with different expressions of FABP4/5 and CD25 have different clinical characteristics (Supplementary Tables S8–S10). Kaplan–Meier survival curve analysis further confirmed that patients with high FABP4/5 expression and high CD25^+^ cell infiltration levels had significantly shorter survival times (Figure 9F, Supplementary Figure S9D). TCGA pan-cancer analysis further showed that *FABP5* expression was significantly increased in cervical cancer and correlated with poor prognosis (Supplementary Figure S9E,F).

## 4. Discussion

By integrating public single-cell transcriptomic data from cervical cancer patients before and after CCRT, multiple independent GEO datasets, and experimental validation including in vitro cell culture and multi-sample immunofluorescence staining, this study characterized the dynamic metabolic and immune landscape of the tumor microenvironment at the single-cell resolution. We revealed global metabolic reprogramming in cervical cancer tissues following CCRT and identified *FABP4/5* as key markers associated with lipid metabolic remodeling in malignant epithelial cells. Furthermore, we demonstrated that *FABP4/5*-high epithelial cells are associated with metabolic remodeling and immunosuppressive differentiation in T cells through enhanced intercellular communication. These findings fill a critical gap in understanding the metabolic–immune crosstalk between tumor and immune cells after CCRT in cervical cancer and provide novel molecular targets for therapeutic intervention.

We acknowledge that the GSE236738 dataset has been utilized in several secondary analyses to characterize the immune microenvironment remodeling induced by CCRT in cervical cancer [25,26,27,28]. While these studies similarly reported an enrichment of immunosuppressive T cell subsets—including regulatory T cells and exhausted T cells—following CCRT, they primarily focused on descriptive immune cell atlas construction or on the prognostic significance of specific immune subpopulations. In contrast, the present study is distinct in three aspects. First, we narrowed the mechanistic focus to *FABP4/5*-mediated lipid metabolic reprogramming in malignant epithelial cells, linking *FABP4/5*-associated metabolic remodeling with immune modulation rather than treating metabolism and immunity as separate descriptive features. Second, through CellChat and NicheNet analyses, we predicted the ligand–receptor axes *PTGER4* and *CXCR4* as potential mediators of epithelial–T cell communication in the context of high *FABP4/5* expression, which represents a mechanistic-level hypothesis extending beyond prior descriptive observations. Third, we validated the clinical relevance of FABP4/5 and CD25^+^ infiltration using multiplex immunofluorescence in an independent cohort of 101 cervical cancer patients and performed siRNA-based functional experiments to verify upstream transcriptional regulation. Nevertheless, the metabolic reprogramming of cervical cancer cells and their interactions with immune cells after CCRT remain poorly characterized at the single-cell resolution. Further investigation is therefore needed to elucidate cellular heterogeneity and metabolic–immune interactions.

Chemoradiotherapy enhances tumor cell survival through multiple mechanisms involving lipid metabolic reprogramming [29]. Fatty acid β-oxidation supplies energy for DNA repair and apoptosis resistance, while lipids serve as signaling molecules activating pro-survival pathways. FABP4 and FABP5 are members of the fatty acid-binding protein family and share substantial structural and functional similarities [30]. Both proteins bind long-chain fatty acids and facilitate their cellular uptake, intracellular transport, and metabolic partitioning. They can also transport lipid signaling molecules to nuclear receptors, thereby potentially influencing downstream lipid metabolic pathways [10]. FABP4 and FABP5 often exhibit co-expression patterns across tissues and functionally compensate for each other in maintaining lipid metabolic homeostasis [31].

Emerging evidence links FABP5 to tumor progression and immune modulation. In triple-negative breast cancer, FABP5 upregulation activates mTOR signaling via ω-6 linoleic acid, enhancing proliferation [12]. In hepatocellular carcinoma, macrophages stimulated by long-chain unsaturated fatty acids promote an immunosuppressive microenvironment through FABP5-PPARγ signaling [13]. In cervical cancer, FABP5-mediated TGF-β signaling contributes to immunosuppressive microenvironment formation, facilitates bidirectional interactions with immunosuppressive cancer-associated fibroblasts, and promotes lymph node metastasis through lipid metabolic reprogramming [15,16,32]. Multiple independent GEO datasets and immunofluorescence staining of 101 cervical cancer patients in this study indicate that FABP4/5 expression is elevated in cervical cancer and is positively correlated with CD25. Our findings extend these observations by demonstrating that epithelial cells with high FABP4/5 expression are associated with active remodeling of T cell metabolism and function, providing new insights into the metabolic–immune axis underlying resistance to concurrent chemoradiotherapy.

This study revealed significant upregulation of *FABP4/5* in malignant epithelial cells of cervical cancer following CCRT, with expression levels positively correlated with the epithelial differentiation trajectory (Figure 2 and Figure 3), suggesting their involvement in malignant transformation. During squamous epithelial malignant progression, altered keratin expression—including upregulation of *KRT5*, *KRT6A*, *KRT13*, and *KRT16*—serves as a key marker of abnormal differentiation [33]. *FABP4/5*-high epithelial cells co-enriched these keratin genes along with proliferation- and differentiation-associated genes (*OLFM4*, *SFN*, *LGALS7*) (Figure 3F,G), further supporting the link between *FABP4/5* and the malignant phenotype.

*FABP4/5* upregulation reflects the metabolic remodeling demands of tumor cells under therapeutic stress. High *FABP4/5* expression was associated with activation of PPAR and adipocytokine signaling pathways, consistent with a potential link between *FABP4/5* expression and epithelial lipid metabolic reprogramming. PPAR pathway activation closely associates with tumor proliferation, survival, and chemoresistance across multiple cancer types [34]. The observed coupling between PPAR pathway activity and *FABP4/5* expression suggests that this axis may contribute to the metabolic adaptation of cervical cancer epithelial cells after CCRT. These findings provide a rationale for further investigating PPAR inhibition in combination with CCRT, although the therapeutic efficacy of this strategy requires experimental validation.

At the transcriptional level, SCENIC analysis identified transcription factors *EHF*, *ELF3*, and *HOXB2* with significantly enhanced activity in *FABP4/5*-high epithelial cells (Figure 4C,D). Using siRNA to knock down these transcription factors, we further confirmed that *EHF* and *HOXB2* positively regulated *FABP4/5*, whereas *ELF3* negatively regulated *FABP4/5* (Figure 4F–H). This apparent discrepancy may reflect the context-dependent nature of transcriptional regulation. *EHF* and *ELF3* are members of the epithelium-specific ETS family, whose transcriptional effects depend on the cellular context and regulatory landscape. EHF has been shown to occupy epithelial enhancer regions and regulate epithelial differentiation programs, consistent with its positive regulation of *FABP4/5* observed in our study [35,36]. In contrast, ELF3 can function as either a transcriptional activator or repressor depending on its target gene; notably, ELF3 was previously shown to activate SPRR2A while repressing KRT4 in cervical epithelial cancer cells [37]. Recent studies further indicate that ELF3 contributes to the maintenance of epithelial identity and restrains epithelial–mesenchymal plasticity [38]. Thus, the inhibitory effect of ELF3 on *FABP4/5* may reflect its context-dependent role in maintaining a specific epithelial transcriptional state. HOXB2 is also relevant to cervical cancer, as its expression increases during cervical carcinogenesis and is associated with HPV infection [39]. Taken together, *EHF* and *HOXB2* may promote the *FABP4/5*-associated transcriptional program, whereas *ELF3* may provide a counter-regulatory mechanism that restrains *FABP4/5* expression. These findings highlight that SCENIC-inferred transcription factor activity reflects a context-dependent regulatory network rather than uniform transcriptional activation or repression.

Tumor epithelial cells actively shape the microenvironment and modulate immune cell function through secreted cytokines, metabolites, and exosomes [40]. CellChat analysis identified *FABP4/5*-high epithelial cells as potential signaling hubs, exhibiting significantly stronger communication with T cells than with other cell types (Figure 5A,B). NicheNet analysis identified key ligand–receptor pairs potentially mediating this interaction, including *PTGER4* and *CXCR4* (Figure 5 and Figure 6). *PTGER4* signaling is known to inhibit T cell proliferation and effector function while promoting Treg differentiation [41,42]; *CXCR4* and its ligand *CXCL12* recruit T cells to the tumor microenvironment and associate with T cell exhaustion [43]. The communication intensity of *PTGER4*-positive and *CXCR4*-positive T cells was significantly higher in strong-communication subgroups (Figure 6F,G), suggesting that these receptors may represent candidate mediators of the observed association between FABP4/5-high epithelial cells and T cell states. However, because these inferences are based on computational prediction rather than direct experimental perturbation of the *PTGER4*/*CXCR4* axes, they should be interpreted as associative and hypothesis-generating rather than conclusive evidence of causal drive.

The metabolic and transcriptional features of T cells with high communication intensity to *FABP4/5*-high epithelial cells further support an immunosuppressive phenotype association. These T cells exhibited reduced expression of effector molecules *GZMB*, *IFNG*, and *PRF1*, alongside metabolic shifts favoring synthetic over catabolic pathways (Figure 6H,I and Figure 7A–D). Notably, high-communication T cells contained significantly higher proportions of CD4^+^ regulatory T cells (Treg), CD4^+^ Tfh-like cells, and CD4^+^ Th17-like cells (Figure 7I). While this pattern is consistent with an immunosuppressive milieu, we caution that CD25 (IL-2RA) was used as a surrogate marker for Treg infiltration in our immunofluorescence cohort. CD25 is also expressed on activated effector T cells upon antigen stimulation; therefore, CD25^+^ cell counts may overestimate true Treg abundance and could partially reflect activated cytotoxic or helper T cell populations. Because FOXP3 and CD4 were not assessed concurrently, interpreting CD25^+^ infiltrates as exclusively immunosuppressive may overestimate the abundance of Tregs. Future studies using multiparameter Treg panels are warranted to further characterize this association.

The prognostic potential of *FABP4* and *FABP5* in cervical cancer warrants attention. Pan-cancer analysis of TCGA data revealed that *FABP5* is upregulated in multiple tumor types (e.g., ACC, HNSC, LUSC), with the most pronounced increase observed in cervical cancer (CESC) (Supplementary Figure S9E), and a trend toward an association between *FABP4* or *FABP5* expression levels and overall prognosis was noted (Supplementary Figure S9F). Through multiplex immunofluorescence and prognostic analysis, the present study further found that patients with high FABP4/5 expression exhibit poor prognosis accompanied by increased infiltration of CD25^+^ cells. Given that concurrent chemoradiotherapy (CCRT) is the cornerstone of treatment for locally advanced cervical cancer, and the present study observed significant changes in *FABP4/5* expression before and after CCRT, the potential value of *FABP4/5* in treatment response assessment and prediction merits further investigation. Future studies in homogeneous CCRT treatment cohorts are needed to elucidate the relationship between *FABP4/5* expression and patient prognosis as well as treatment sensitivity.

## 5. Conclusions

This study suggests that CCRT is associated with *FABP4/5*-mediated lipid metabolic reprogramming in cervical cancer epithelial cells, which may in turn correlate with immunosuppressive crosstalk with T cells through the *PTGER4* and *CXCR4* signaling axes. This enhanced intercellular communication appears to coincide with T cell metabolic remodeling and an increased prevalence of regulatory T cells, potentially facilitating immune evasion and possibly contributing to treatment resistance. Accordingly, FABP4/5 and its downstream effectors may warrant further investigation as potential targets for immunometabolic intervention to improve CCRT outcomes and reduce recurrence in locally advanced cervical cancer.

## 6. Study Limitations

This study has several limitations. First, the single-cell transcriptomic analysis was based on paired samples from only three cervical cancer patients. Although batch effect correction was applied during data integration, the limited sample size may not fully capture inter-patient heterogeneity, and the cell–cell communication findings should be interpreted as exploratory and require validation in larger cohorts. Second, while siRNA knockdown preliminarily validated the regulatory effects of *EHF*, *ELF3*, and *HOXB2* on *FABP4/5*, the detailed molecular mechanisms (e.g., direct promoter binding or chromatin remodeling) remain to be fully elucidated through functional assays. Third, CD25 (IL-2RA) was used as a surrogate marker for immunosuppressive T cell infiltration; as CD25 can also be expressed on activated effector T cells, future studies incorporating FOXP3-based validation are warranted to refine Treg quantification. Fourth, the clinical correlation analysis was performed retrospectively in a single-center cohort, and the predictive value of FABP4/5 for CCRT response and long-term survival requires prospective, multi-center validation.

## Data Availability

The single-cell transcriptomic data used in this study originated from the public gene expression database (Gene Expression Omnibus, GEO) datasets GSE236738, GSE9750, GSE52903 and GSE39001.

## Supplementary Materials

The following supporting information can be downloaded at: https://www.mdpi.com/article/10.3390/biomedicines14092072/s1.

## Abbreviations

The following abbreviations are used in this manuscript:

ACC	Adrenocortical Carcinoma
CCRT	Concurrent chemoradiotherapy
CESC	Cervical squamous cell carcinoma
DC	Dendritic cell
DEGs	Differentially expressed genes
FABPs	Fatty acid-binding proteins
GO	Gene Ontology
GSEA	Gene set enrichment analysis
HNSC	Head and Neck Squamous Cell Carcinoma
HSC-G-CSF	Hematopoietic stem cells
KEGG	Kyoto Encyclopedia of Genes and Genomes
LUSC	Lung Squamous Cell Carcinoma
NK	Natural killer
PCA	Principal component analysis
TCGA	The Cancer Genome Atlas
TF	Transcription factor
TME	Tumor microenvironment
