# Supplementary material for: FABP4/5-Mediated Lipid Reprogramming Is Associated with Immunosuppressive T Cell Crosstalk in Cervical Cancer After Chemoradiotherapy
Source: Biomedicines. 2026 Sep 15;14(9):2072. doi: 10.3390/biomedicines14092072 (PMC13604168; doi:10.3390/biomedicines14092072)
Supplement: Supplementary file 1 [file biomedicines-14-02072-s001.zip › Supplementary Figures.pdf]

Figure S1

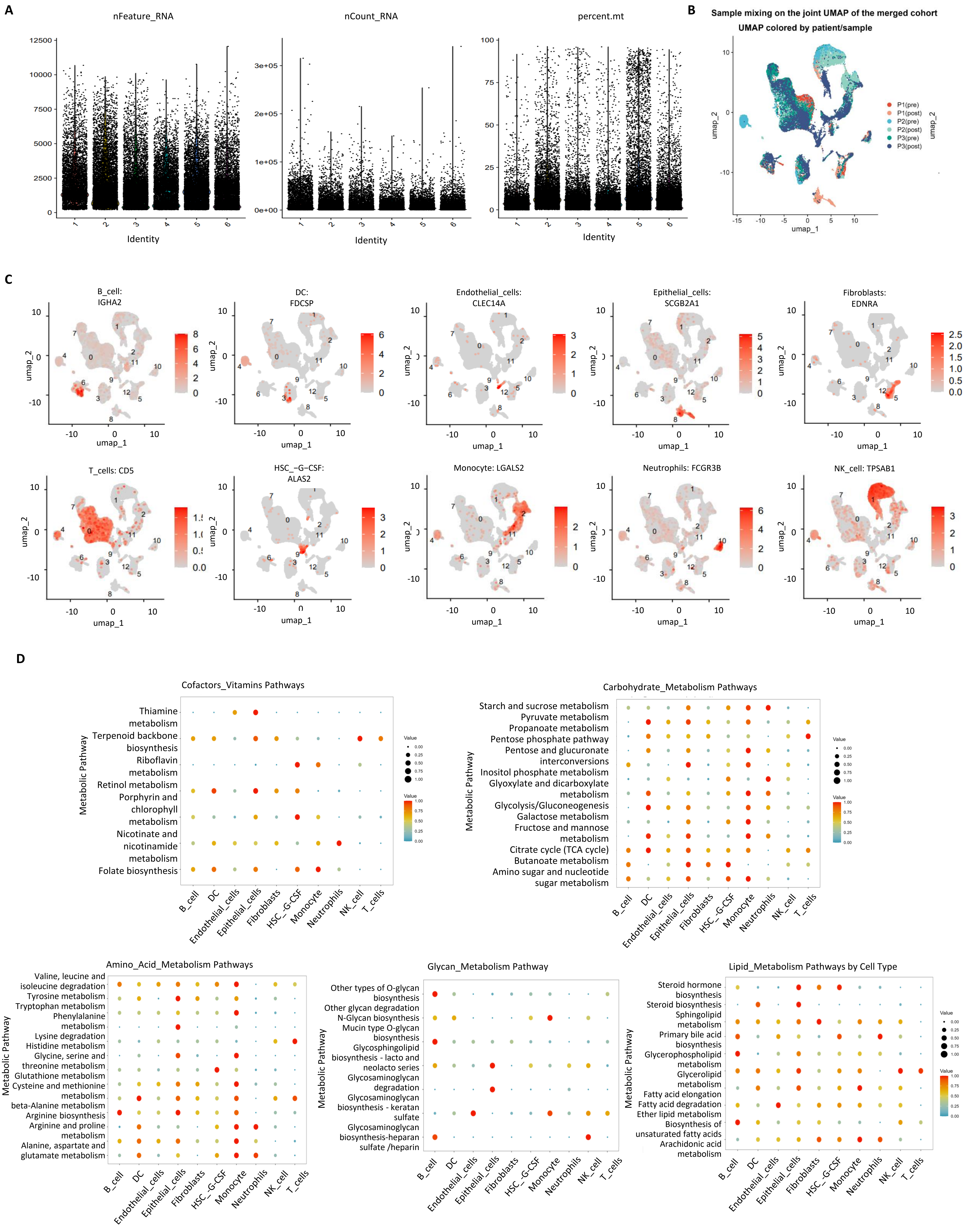

A
nFeature_RNA
nCount_RNA
percent.mt
Identity
B
Sample mixing on the joint UMAP of the merged cohort
UMAP colored by patient/sample
P1(pre)
P1(post)
P2(pre)
P2(post)
P3(pre)
P3(post)
umap_1
umap_2
C
B_cell: IGHA2
DC: FDCSP
Endothelial_cells: CLEC14A
Epithelial_cells: SCGB2A1
Fibroblasts: EDNRA
T_cells: CD5
HSC_-G-CSF: ALAS2
Monocyte: LGALS2
Neutrophils: FCGR3B
NK_cell: TPSAB1
D
Cofactors_Vitamins Pathways
Thiamine metabolism
Terpenoid backbone biosynthesis
Riboflavin metabolism
Retinol metabolism
Porphyrin and chlorophyll metabolism
Nicotinate and nicotinamide metabolism
Folate biosynthesis
Carbohydrate_Metabolism Pathways
Starch and sucrose metabolism
Pyruvate metabolism
Propanoate metabolism
Pentose phosphate pathway
Pentose and glucuronate interconversions
Inositol phosphate metabolism
Glyoxylate and dicarboxylate metabolism
Glycolysis/Gluconeogenesis
Galactose metabolism
Fructose and mannose metabolism
Citrate cycle (TCA cycle)
Butanoate metabolism
Amino sugar and nucleotide sugar metabolism
Amino_Acid_Metabolism Pathways
Valine, leucine and isoleucine degradation
Tyrosine metabolism
Tryptophan metabolism
Phenylalanine metabolism
Lysine degradation
Histidine metabolism
Glycine, serine and threonine metabolism
Glutathione metabolism
Cysteine and methionine metabolism
beta-Alanine metabolism
Arginine biosynthesis
Arginine and proline metabolism
Alanine, aspartate and glutamate metabolism
Glycan_Metabolism Pathway
Other types of O-glycan biosynthesis
Other glycan degradation
N-Glycan biosynthesis
Mucin type O-glycan biosynthesis
Glycosphingolipid biosynthesis - lacto and neolacto series
Glycosaminoglycan degradation
Glycosaminoglycan biosynthesis - keratan sulfate
Glycosaminoglycan biosynthesis-heparan sulfate /heparin
Lipid_Metabolism Pathways by Cell Type
Steroid hormone biosynthesis
Steroid biosynthesis
Sphingolipid metabolism
Primary bile acid biosynthesis
Glycerophospholipid metabolism
Glycerolipid metabolism
Fatty acid elongation
Fatty acid degradation
Ether lipid metabolism
Biosynthesis of unsaturated fatty acids
Arachidonic acid metabolism
Metabolic Pathway
Value
B_cell
DC
Endothelial_cells
Epithelial_cells
Fibroblasts
HSC_G-CSF
Monocyte
Neutrophils
NK_cell
T_cells

Supplementary Figure S1. Single-cell RNA sequencing data quality control and cellular feature analysis. (a) Violin plots showing the distribution of three key quality control metrics across different samples (Identity) in the single-cell RNA sequencing (scRNA-seq) data. (b) Joint UMAP shows the sample composition of each annotated cell type. (c) Expression distribution of specific marker genes across different cell types. (d) Bubble plot of metabolic pathway activity scores stratified by major cell types.

Figure S2

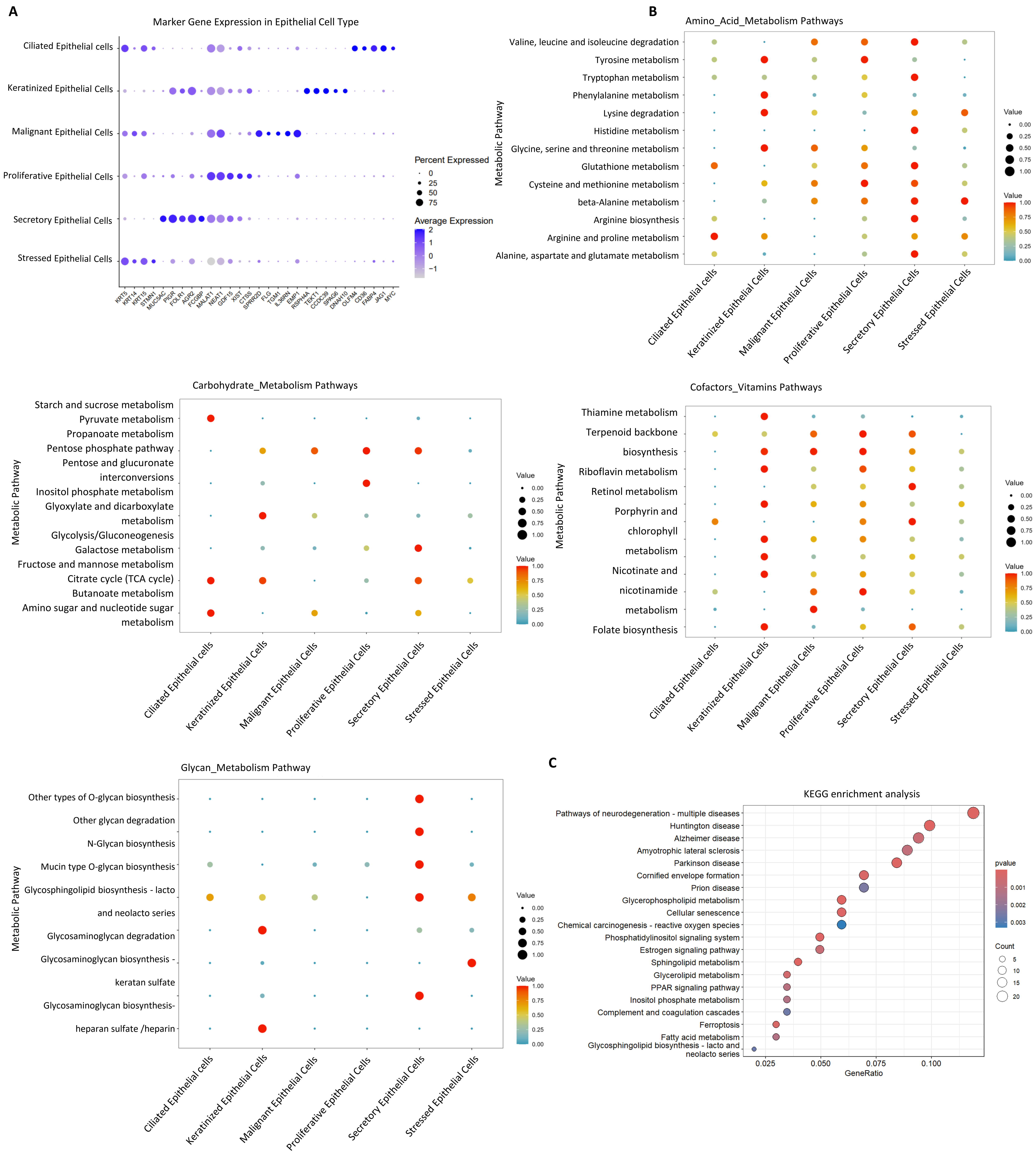

Supplementary Figure S2. Gene expression signatures and functional enrichment analysis of epithelial cell subpopulations. (a) Expression profiles of characteristic marker genes across distinct epithelial cell subpopulations. (b) Bubble plot of metabolic pathway activity scores stratified by major epithelial cell types. (c) Bar plot of KEGG enrichment analysis for identified differentially expressed genes.

Figure S3

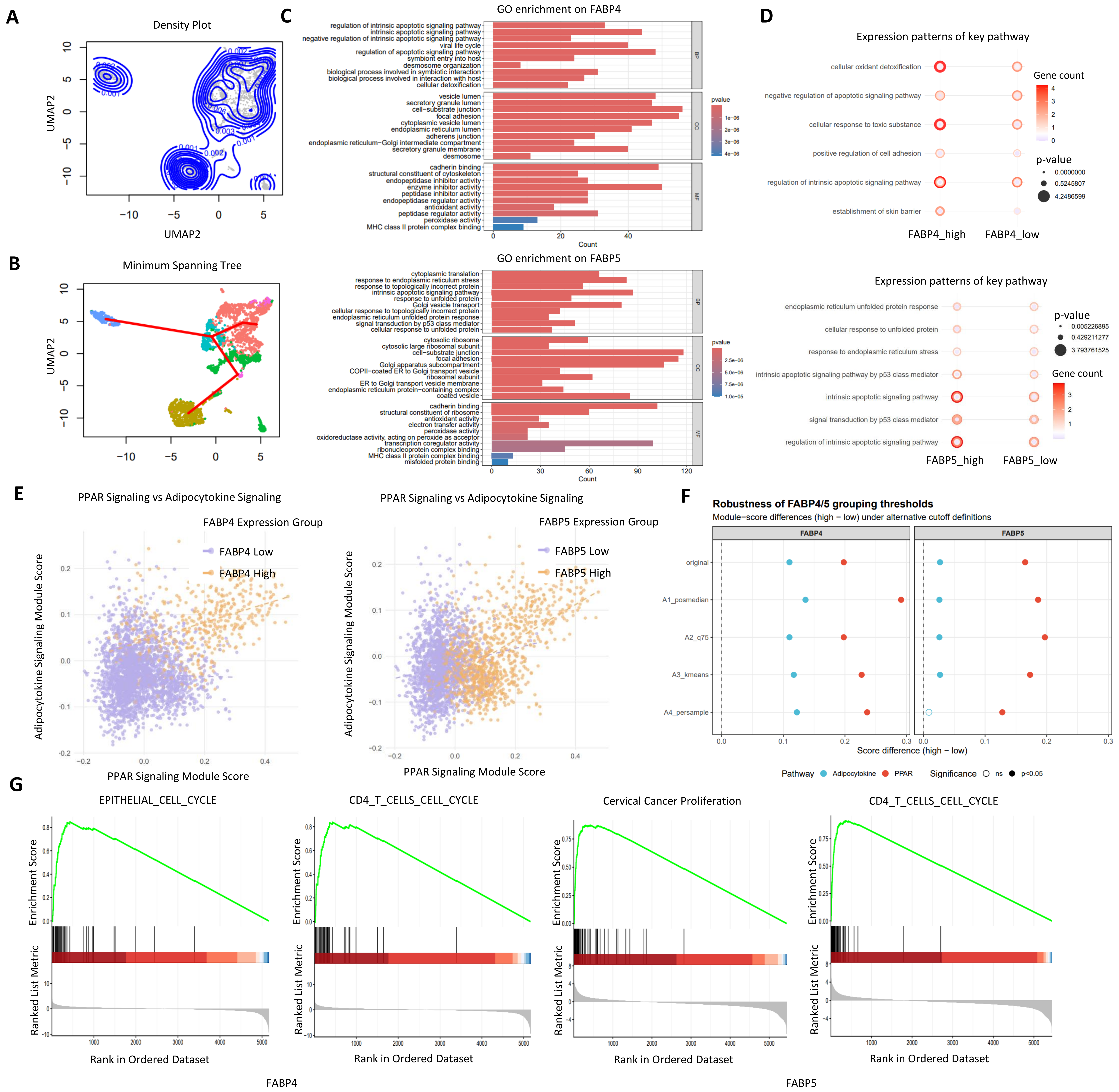

Supplementary Figure S3. Characterization and regulatory mechanisms of FABP4- and FABP5-associated cell subpopulations. (a) UMAP visualization showing cell population distribution density using density contours. (b) Minimum spanning tree (MST) graph illustrating developmental or differentiation relationships among different cell subpopulations. (c) Bar plots of GO enrichment analysis showing significant enrichment of biological processes in comparisons of differentially expressed genes for FABP4 (top) and FABP5 (bottom). (d) Dot plots of key pathways showing differentially enriched biological pathways in FABP4-high versus FABP4-low (top) and FABP5-high versus FABP5-low (bottom) cell populations. (e) Scatter plots comparing module scores of PPAR signaling and adipocytokine signaling pathways between epithelial subpopulations with high versus low FABP4/5 expression. (f) The original FABP4/5 high/low stratification (global median split of log-normalized expression across all epithelial cells) was re-derived under four alternative cutoff schemes: median of detection-positive cells, global 75th percentile, k-means clustering (k = 2), and per-sample median. For each scheme, differences in PPAR signaling and adipocytokine signaling module scores between high- and low-expression groups were recalculated. (g) Gene set enrichment analysis (GSEA) of epithelial cell cycle, T cell cycle, and cervical cancer proliferation-related gene sets in differentially expressed genes between high- and low-expression subgroups.

Figure S4

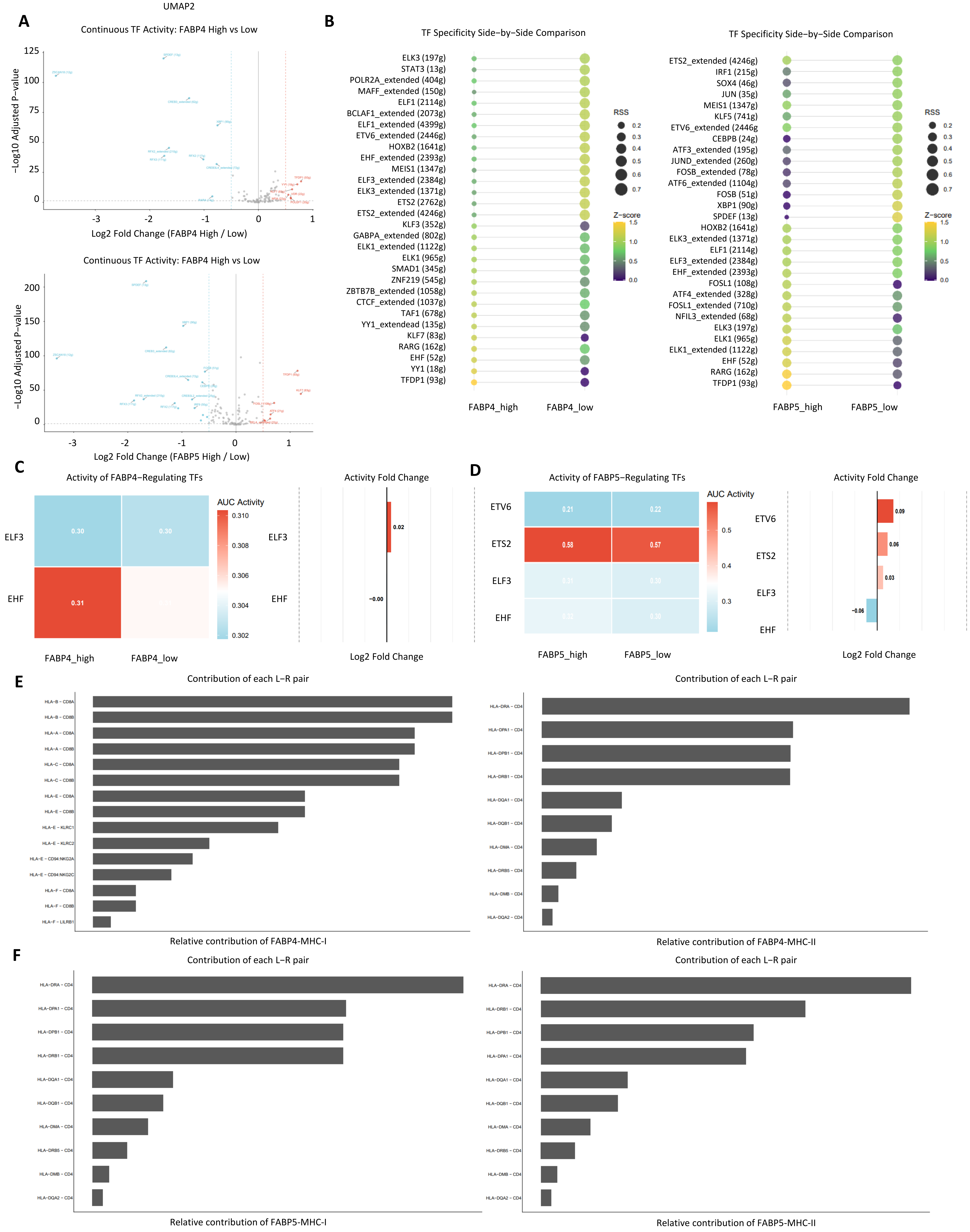
UMAP2
A
Continuous TF Activity: FABP4 High vs Low
−Log10 Adjusted P−value
Log2 Fold Change (FABP4 High / Low)
Continuous TF Activity: FABP4 High vs Low
−Log10 Adjusted P−value
Log2 Fold Change (FABP5 High / Low)
B
TF Specificity Side−by−Side Comparison
ELK3 (197g)
STAT3 (13g)
POLR2A_extended (404g)
MAFF_extended (150g)
ELF1 (2114g)
BCLAF1_extended (2073g)
ELF1_extended (4399g)
ETV6_extended (2446g)
HOXB2 (1641g)
EHF_extended (2393g)
MEIS1 (1347g)
ELF3_extended (2384g)
ELK3_extended (1371g)
ETS2 (2762g)
ETS2_extended (4246g)
KLF3 (352g)
GABPA_extended (802g)
ELK1_extended (1122g)
ELK1 (965g)
SMAD1 (345g)
ZNF219 (545g)
ZBTB7B_extended (1058g)
CTCF_extended (1037g)
TAF1 (678g)
YY1_extendead (135g)
KLF7 (83g)
RARG (162g)
EHF (52g)
YY1 (18g)
TFDP1 (93g)
FABP4_high
FABP4_low
RSS
Z−score
TF Specificity Side−by−Side Comparison
ETS2_extended (4246g)
IRF1 (215g)
SOX4 (46g)
JUN (35g)
MEIS1 (1347g)
KLF5 (741g)
ETV6_extended (2446g
CEBPB (24g)
ATF3_extended (195g)
JUND_extended (260g)
FOSB_extended (78g)
ATF6_extended (1104g)
FOSB (51g)
XBP1 (90g)
SPDEF (13g)
HOXB2 (1641g)
ELK3_extended (1371g)
ELF1 (2114g)
ELF3_extended (2384g)
EHF_extended (2393g)
FOSL1 (108g)
ATF4_extended (328g)
FOSL1_extended (710g)
NFIL3_extended (68g)
ELK3 (197g)
ELK1 (965g)
ELK1_extended (1122g)
EHF (52g)
RARG (162g)
TFDP1 (93g)
FABP5_high
FABP5_low
RSS
Z−score
C
Activity of FABP4−Regulating TFs
ELF3
EHF
FABP4_high
FABP4_low
AUC Activity
Activity Fold Change
Log2 Fold Change
D
Activity of FABP5−Regulating TFs
ETV6
ETS2
ELF3
EHF
FABP5_high
FABP5_low
AUC Activity
Activity Fold Change
Log2 Fold Change
E
Contribution of each L−R pair
HLA−B − CD8A
HLA−B − CD8B
HLA−A − CD8A
HLA−A − CD8B
HLA−C − CD8A
HLA−C − CD8B
HLA−E − CD8A
HLA−E − CD8B
HLA−E − KLRC1
HLA−E − KLRC2
HLA−E − CD94:NKG2A
HLA−E − CD94:NKG2C
HLA−F − CD8A
HLA−F − CD8B
HLA−F − LILRB1
Relative contribution of FABP4-MHC-I
Contribution of each L−R pair
HLA−DRA − CD4
HLA−DPA1 − CD4
HLA−DPB1 − CD4
HLA−DRB1 − CD4
HLA−DQA1 − CD4
HLA−DQB1 − CD4
HLA−DMA − CD4
HLA−DRB5 − CD4
HLA−DMB − CD4
HLA−DQA2 − CD4
Relative contribution of FABP4-MHC-II
F
Contribution of each L−R pair
HLA−DRA − CD4
HLA−DPA1 − CD4
HLA−DPB1 − CD4
HLA−DRB1 − CD4
HLA−DQA1 − CD4
HLA−DQB1 − CD4
HLA−DMA − CD4
HLA−DRB5 − CD4
HLA−DMB − CD4
HLA−DQA2 − CD4
Relative contribution of FABP5-MHC-I
Contribution of each L−R pair
HLA−DRA − CD4
HLA−DRB1 − CD4
HLA−DPB1 − CD4
HLA−DPA1 − CD4
HLA−DQA1 − CD4
HLA−DQB1 − CD4
HLA−DMA − CD4
HLA−DRB5 − CD4
HLA−DMB − CD4
HLA−DQA2 − CD4
Relative contribution of FABP5-MHC-II

Supplementary Figure S4. Differential expression profiles, transcription factor activities, and immune-related ligand-receptor interactions of FABP4/5-high versus FABP4/5-low epithelial cell subpopulations. (a) Volcano plots of differentially expressed genes in FABP4-high versus FABP4-low (top) and FABP5-high versus FABP5-low (bottom) comparisons. (b) Transcription factor (TF) specificity comparison plots for FABP4-high versus FABP4-low (left) and FABP5-high versus FABP5-low (right) cell populations. (c, d) Heatmaps and log2 fold change plots showing the activity of transcription factors (ELF3, EHF) regulating FABP4 and FABP5. (e) Bar plots showing the relative contributions of individual ligand-receptor (L-R) pairs associated with FABP4-MHC-I (left) and FABP4-MHC-II (right) interactions. (f) Bar plots showing the relative contributions of individual ligand-receptor (L-R) pairs associated with FABP5-MHC-I (left) and FABP5-MHC-II (right) interactions.

Figure S5

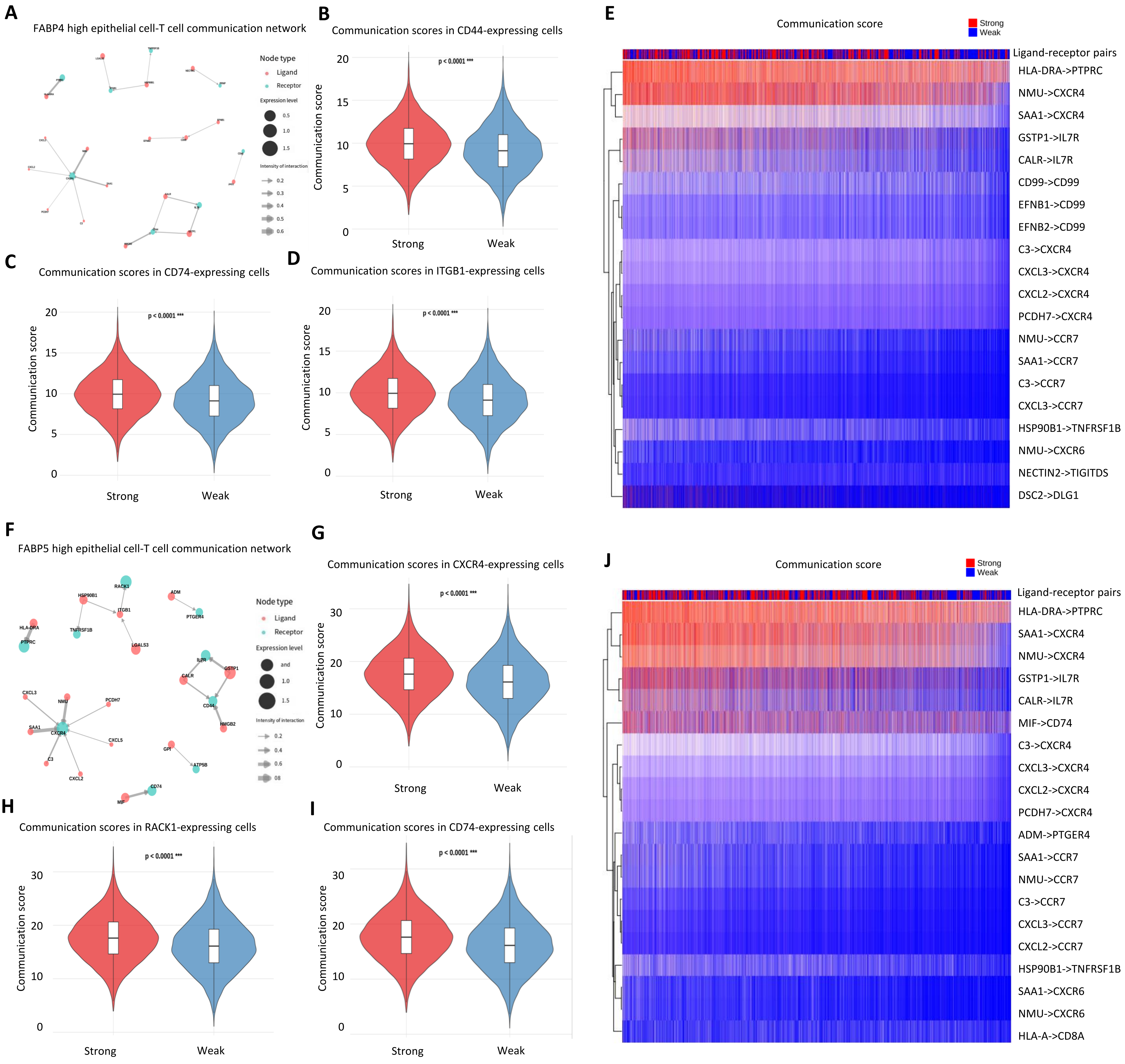

Supplementary Figure S5. Analysis of differences in communication networks and intensities between epithelial cells and T cells with high FABP4/5 expression. (a) Schematic diagram of the communication network between FABP4-high epithelial cells and T cells. (b, c, d) Violin plots showing communication scores of cells expressing CD44 (b), CD74 (c), and ITGB1 (d) in "strong" versus "weak" communication states (FABP4 context). (e) Heatmap of communication scores for individual ligand-receptor (LR) pairs between strong and weak T cell subsets (FABP4 context). (f) Schematic diagram of the communication network between FABP5-high epithelial cells and T cells. (g, h, i) Violin plots showing communication scores of cells expressing CXCR4 (g), RACK1 (h), and CD74 (i) in "strong" versus "weak" communication states (FABP5 context). (j) Heatmap of communication scores for individual ligand-receptor (L-R) pairs between strong and weak T cell subsets (FABP5 context).

Figure S6

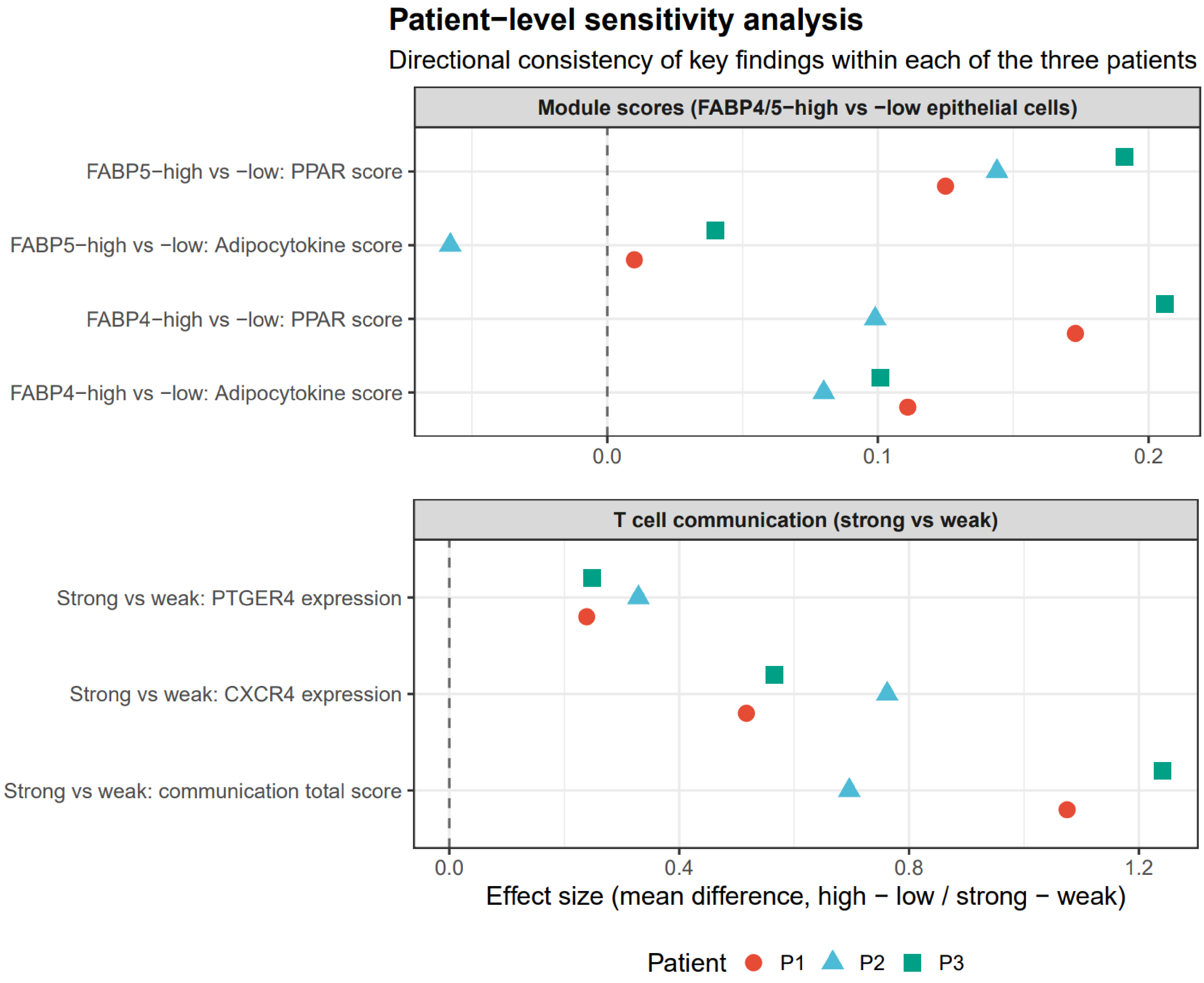

Supplementary Figure S6. Patient-level sensitivity analysis of the core single-cell findings. Differences in PPAR and adipocytokine signaling module scores between FABP4/5-high and -low epithelial cells were directionally consistent with the pooled analysis in 11 of 12 patient-level comparisons (the only exception was FABP5–adipocytokine in patient P2, with a small absolute difference). Differences in communication intensity, PTGER4 expression, and CXCR4 expression between strong- and weak-communication T cells were consistent in all 9 of 9 patient-level comparisons (all $p < 10^{-12}$). Each point represents one patient-level comparison; the dashed line indicates zero difference.

Figure S7

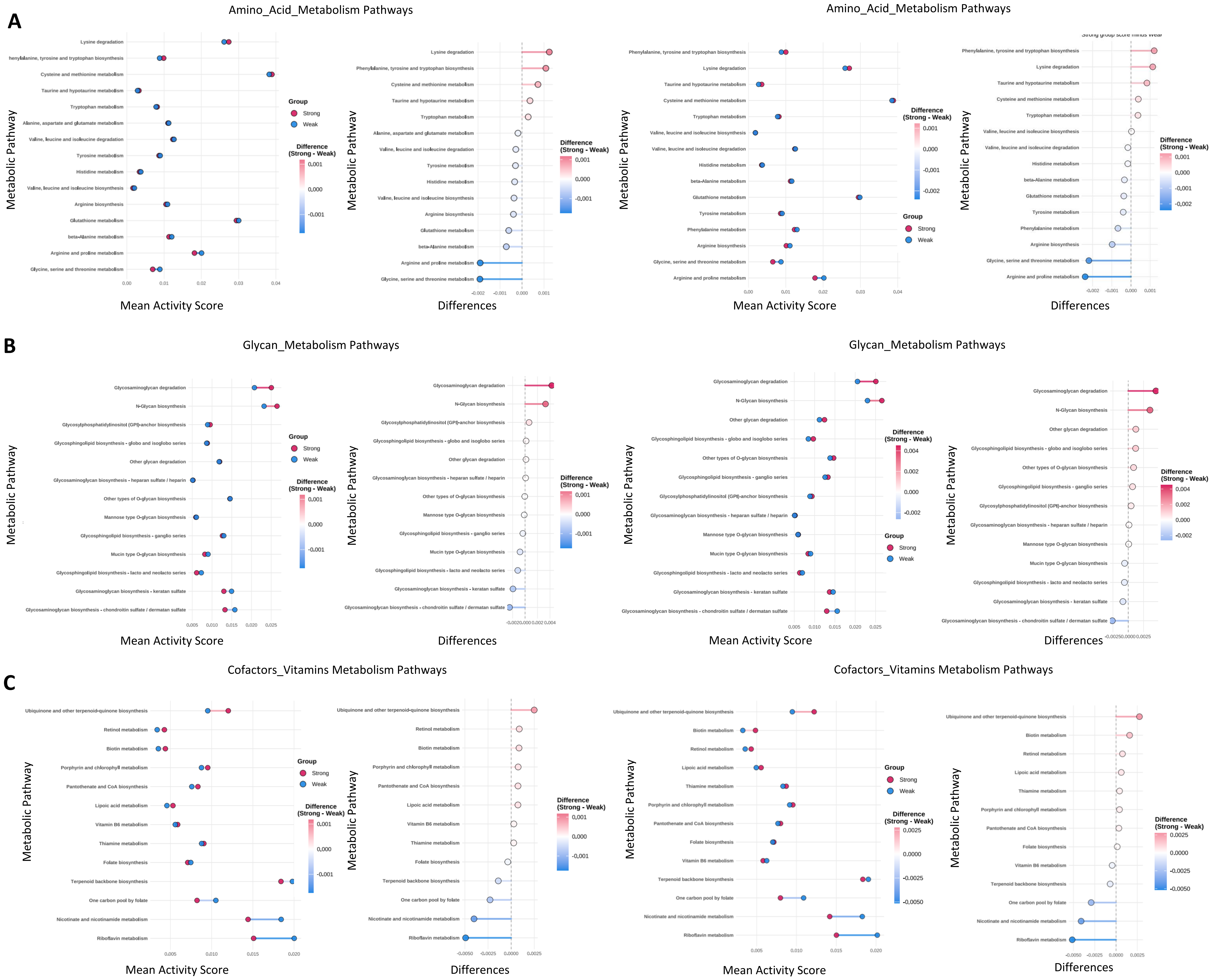

Supplementary Figure S7. T cell metabolic pathway activities by FABP4/5 communication intensity. (a-b) Violin plots comparing pathway activity scores of Amino Acid Metabolism (a), Glycan Metabolism (b), and Metabolism of Cofactors and Vitamins (c) between strong and weak communication T cell subsets stratified by communication intensity with FABP4-high (left panels) or FABP5-high (right panels) epithelial cells.

Figure S8

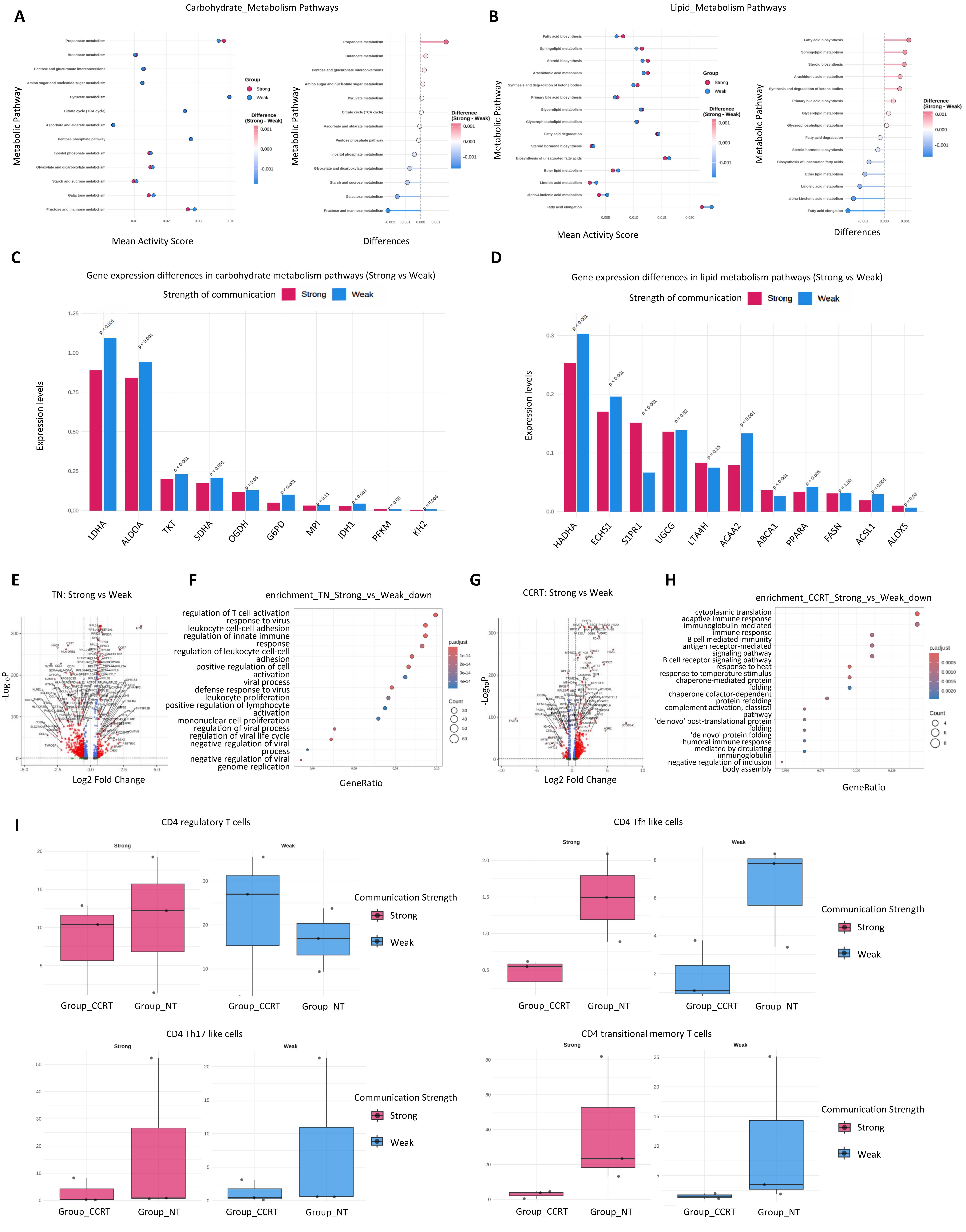
A
Carbohydrate_Metabolism Pathways
Metabolic Pathway
Propanoate metabolism
Butanoate metabolism
Pentose and glucuronate interconversions
Amino sugar and nucleotide sugar metabolism
Pyruvate metabolism
Citrate cycle (TCA cycle)
Ascorbate and aldarate metabolism
Pentose phosphate pathway
Inositol phosphate metabolism
Glyoxylate and dicarboxylate metabolism
Starch and sucrose metabolism
Galactose metabolism
Fructose and mannose metabolism
Group
Strong
Weak
Difference (Strong - Weak)
Mean Activity Score
Differences
B
Lipid_Metabolism Pathways
Fatty acid biosynthesis
Sphingolipid metabolism
Steroid biosynthesis
Arachidonic acid metabolism
Synthesis and degradation of ketone bodies
Primary bile acid biosynthesis
Glycerolipid metabolism
Glycerophospholipid metabolism
Fatty acid degradation
Steroid hormone biosynthesis
Biosynthesis of unsaturated fatty acids
Ether lipid metabolism
Linoleic acid metabolism
alpha-Linolenic acid metabolism
Fatty acid elongation
C
Gene expression differences in carbohydrate metabolism pathways (Strong vs Weak)
Strength of communication
Strong
Weak
Expression levels
LDHA
ALDOA
TKT
SDHA
OGDH
G6PD
MPI
IDH1
PFKM
KH2
D
Gene expression differences in lipid metabolism pathways (Strong vs Weak)
HADHA
ECHS1
S1PR1
UGGG
LTA4H
ACAA2
ABCA1
PPARA
FASN
ACSL1
ALOX5
E
TN: Strong vs Weak
$-Log_{10}P$
Log2 Fold Change
F
enrichment_TN_Strong_vs_Weak_down
regulation of T cell activation
response to virus
leukocyte cell-cell adhesion
regulation of innate immune response
regulation of leukocyte cell-cell adhesion
positive regulation of cell activation
viral process
defense response to virus
leukocyte proliferation
positive regulation of lymphocyte activation
mononuclear cell proliferation
regulation of viral process
regulation of viral life cycle
negative regulation of viral process
negative regulation of viral genome replication
p.adjust
Count
GeneRatio
G
CCRT: Strong vs Weak
H
enrichment_CCRT_Strong_vs_Weak_down
cytoplasmic translation
adaptive immune response
immunoglobulin mediated immune response
B cell mediated immunity
antigen receptor-mediated signaling pathway
B cell receptor signaling pathway
response to heat
response to temperature stimulus
chaperone-mediated protein folding
chaperone cofactor-dependent protein refolding
complement activation, classical pathway
'de novo' post-translational protein folding
'de novo' protein folding
humoral immune response mediated by circulating immunoglobulin
negative regulation of inclusion body assembly
I
CD4 regulatory T cells
CD4 Tfh like cells
CD4 Th17 like cells
CD4 transitional memory T cells
Strong
Weak
Group_CCRT
Group_NT
Communication Strength

Supplementary Figure S8. Metabolic reprogramming, transcriptomic alterations, and phenotypic heterogeneity of strong versus weak T cell subsets (FABP4 context) in cervical cancer. (A, B) Violin plots comparing lipid metabolism (A) and carbohydrate metabolism (B) pathway activity scores between strong and weak communication T cell subsets stratified by communication intensity with FABP4-high epithelial cells. (C, D) Bar plots showing expression differences of glycolysis and energy metabolism-related genes (C) and fatty acid metabolism-related genes (D) between strong and weak communication T cell subsets. (E, F) Volcano plot (E) and KEGG pathway enrichment analysis (F) of differentially expressed genes (DEGs) between strong and weak communication T cell subsets before treatment (TN). (G, H) Volcano plot (G) and KEGG pathway enrichment analysis (H) of differentially expressed genes (DEGs) between strong and weak communication T cell subsets after treatment (CCRT). (I) Proportional distribution of CD4+ regulatory T cells, CD4+ Tfh-like cells, CD4+ Th17-like cells, and CD4+ transitional memory T cells between strong and weak communication T cell subsets.

Figure S9

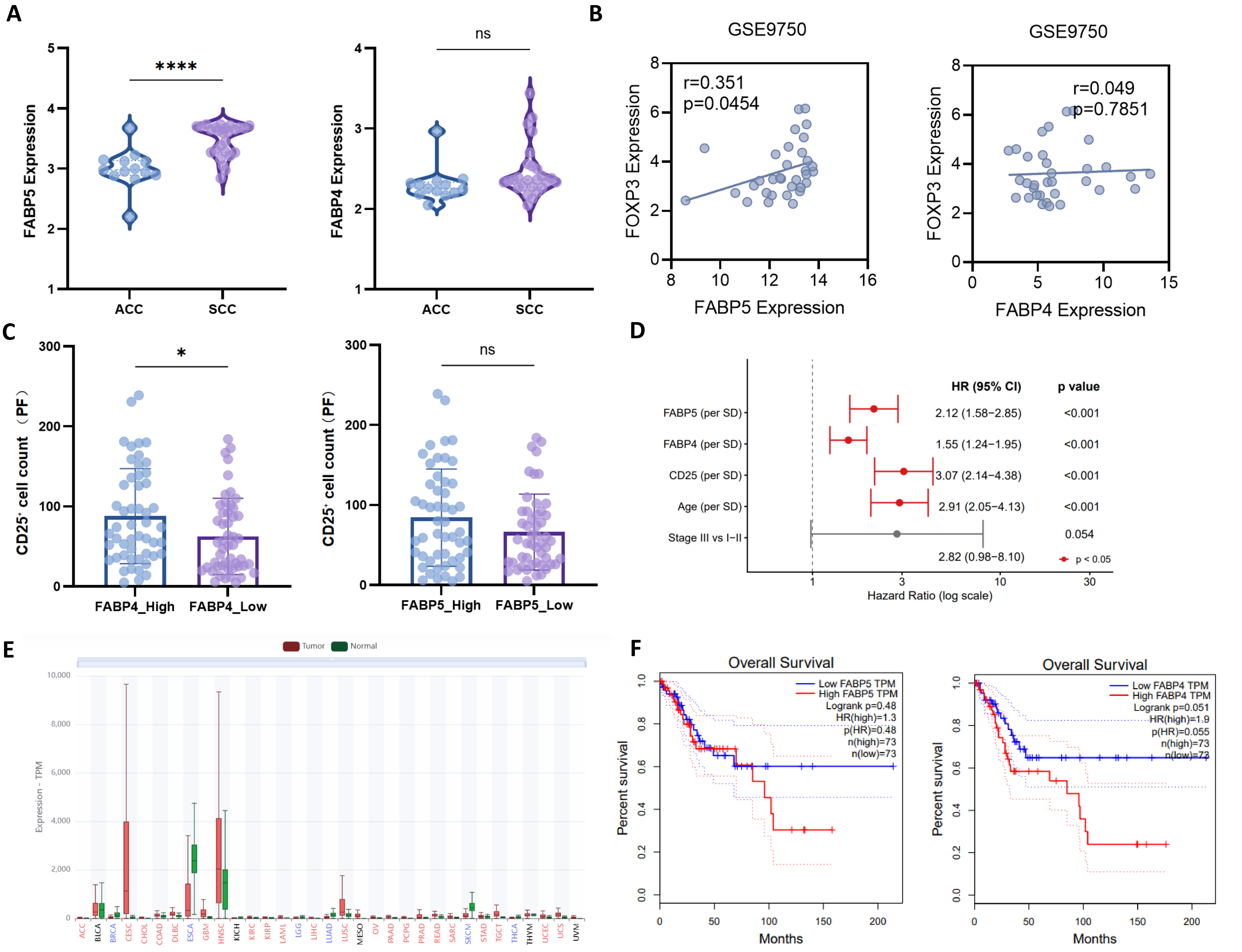

Supplementary Figure S9. Validation of FABP4/5 expression, immune correlation, and prognostic value in public databases. (a) Comparison of FABP4/5 expression levels in ACC and SCC cervical cancer tissues in the GSE275914 dataset (independent t-test). (b) Correlation analysis between FOXP3 and FABP4/5 expression levels in the GSE9750 dataset. (c) Comparison of $CD25^{+}$ cell infiltration levels between high (n=51) and low (n=50) FABP4/5 expression groups using independent t-test. (d) The results of the Cox proportional hazards model were analyzed by univariate analysis. (e) Bar plot showing FABP5 expression levels in tumor versus normal tissues across pan-cancer types. (f) Kaplan-Meier survival curves based on the TCGA cervical cancer database: left panel, overall survival comparison between patients with high and low FABP5 expression; right panel, overall survival comparison between patients with high and low FABP4 expression. Both curves were generated using the GEPIA website.
