# Supplementary material for: FABP4/5-Mediated Lipid Reprogramming Is Associated with Immunosuppressive T Cell Crosstalk in Cervical Cancer After Chemoradiotherapy
Source: Biomedicines. 2026 Sep 15;14(9):2072. doi: 10.3390/biomedicines14092072 (PMC13604168; doi:10.3390/biomedicines14092072)
Supplement: Supplementary file 1 [file biomedicines-14-02072-s001.zip › Supplementary Table S1.pdf]

Table S1. Paired analysis of FABP4, FABP5 and CD25 expression in cervical cancer and adjacent normal tissues.

Variable	Overall, N = 26[1]	Peritumoral_tissue, N = 13[1]	tumor, N = 13[1]	p-value[2]
**FABP4**	594 (80, 3,749)	923 (162, 4,885)	336 (72, 1,200)	0.9970
**FABP5**	6,724 (256, 16,438)	5,357 (215, 8,453)	12,105 (352, 43,784)	0.0479
**CD25**	88 (61, 138)	72 (59, 97)	97 (75, 192)	0.0198

[1]Median (IQR)

[2]Wilcoxon signed-rank test (paired test, paired per patient)
