# Supplementary material for: FABP4/5-Mediated Lipid Reprogramming Is Associated with Immunosuppressive T Cell Crosstalk in Cervical Cancer After Chemoradiotherapy
Source: Biomedicines. 2026 Sep 15;14(9):2072. doi: 10.3390/biomedicines14092072 (PMC13604168; doi:10.3390/biomedicines14092072)
Supplement: Supplementary file 1 [file biomedicines-14-02072-s001.zip › Supplementary Table S10.pdf]

Variable	Overall, N = 101[1]	CD25_High, N = 51[1]	CD25_Low, N = 50[1]	p-value[2]
**FABP5**	30,806 (5,701, 52,355)	40,725 (11,624, 76,514)	24,216 (2,995, 41,207)	0.025
**FABP4**	1,994 (374, 10,323)	2,745 (676, 17,519)	1,120 (347, 4,781)	0.080
**CD25**	60 (30, 102)	102 (84, 154)	30 (20, 41)	<0.001
**Survival_status(Dead)**	29 (29%)	24 (47%)	5 (10%)	<0.001
**Age**	46 (41, 53)	48 (42, 57)	45 (39, 48)	0.013
**Pathological_type**				0.11
AC	3 (3.0%)	3 (5.9%)	0 (0%)	
ASC	4 (4.0%)	1 (2.0%)	3 (6.0%)	
ASCUS	15 (15%)	5 (9.8%)	10 (20%)	
SCC	79 (78%)	42 (82%)	37 (74%)	
**Histotype**				0.4
Ⅰ	16 (16%)	6 (12%)	10 (20%)	
Ⅰ-Ⅱ	5 (5.0%)	3 (5.9%)	2 (4.0%)	
Ⅱ	7 (6.9%)	5 (9.8%)	2 (4.0%)	
Ⅱ-Ⅲ	10 (9.9%)	7 (14%)	3 (6.0%)	
Ⅲ	63 (62%)	30 (59%)	33 (66%)	
**T**				<0.001
T1	55 (54%)	17 (33%)	38 (76%)	
T2	28 (28%)	22 (43%)	6 (12%)	
T3	17 (17%)	11 (22%)	6 (12%)	
T4	1 (1.0%)	1 (2.0%)	0 (0%)	
**N**				0.2
N0	84 (83%)	40 (78%)	44 (88%)	
N1	17 (17%)	11 (22%)	6 (12%)	
**M**				
M0	101 (100%)	51 (100%)	50 (100%)	

**Clinical_stage**				<0.001
1	55 (54%)	17 (33%)	38 (76%)	
2	28 (28%)	22 (43%)	6 (12%)	
3	18 (18%)	12 (24%)	6 (12%)	
**Recurrence**	35 (35%)	28 (55%)	7 (14%)	<0.001
**DFS**	69 (45, 79)	52 (36, 76)	72 (67, 80)	0.004

[1]Median (IQR); n (%)

[2]Wilcoxon rank sum test; Pearson's Chi-squared test; Fisher's exact test
