# Supplementary material for: FABP4/5-Mediated Lipid Reprogramming Is Associated with Immunosuppressive T Cell Crosstalk in Cervical Cancer After Chemoradiotherapy
Source: Biomedicines. 2026 Sep 15;14(9):2072. doi: 10.3390/biomedicines14092072 (PMC13604168; doi:10.3390/biomedicines14092072)
Supplement: Supplementary file 1 [file biomedicines-14-02072-s001.zip › Supplementary Table S2.pdf]

Table S2. Association between Recurrence Status and Clinicopathological Characteristics

Variable	Overall, N = 101[1]	Non-recurred, N = 66[1]	Recurred, N = 35[1]	p-value[2]
**FABP5**	30,806 (5,701, 52,355)	24,938 (4,284, 44,172)	38,153 (10,794, 107,716)	0.018
**FABP4**	1,994 (374, 10,323)	908 (190, 4,238)	10,323 (2,130, 31,590)	<0.001
**CD25**	60 (30, 102)	44 (24, 81)	126 (62, 162)	<0.001
**Survival_status(Dead)**	29 (29%)	0 (0%)	29 (83%)	<0.001
**Age**	46 (41, 53)	44 (39, 47)	55 (47, 62)	<0.001
**Pathological_type**				0.005
AC	3 (3.0%)	0 (0%)	3 (8.6%)	
ASC	4 (4.0%)	3 (4.5%)	1 (2.9%)	
ASCUS	15 (15%)	14 (21%)	1 (2.9%)	
SCC	79 (78%)	49 (74%)	30 (86%)	
**Histotype**				0.12
Ⅰ	16 (16%)	14 (21%)	2 (5.7%)	
Ⅰ-Ⅱ	5 (5.0%)	2 (3.0%)	3 (8.6%)	
Ⅱ	7 (6.9%)	6 (9.1%)	1 (2.9%)	
Ⅱ-Ⅲ	10 (9.9%)	6 (9.1%)	4 (11%)	
Ⅲ	63 (62%)	38 (58%)	25 (71%)	
**T**				<0.001
T1	55 (54%)	48 (73%)	7 (20%)	
T2	28 (28%)	11 (17%)	17 (49%)	
T3	17 (17%)	7 (11%)	10 (29%)	
T4	1 (1.0%)	0 (0%)	1 (2.9%)	
**N**				0.004
N0	84 (83%)	60 (91%)	24 (69%)	
N1	17 (17%)	6 (9.1%)	11 (31%)	
**M**				

M0	101 (100%)	66 (100%)	35 (100%)	
**Clinical_stage**				<0.001
1	55 (54%)	48 (73%)	7 (20%)	
2	28 (28%)	11 (17%)	17 (49%)	
3	18 (18%)	7 (11%)	11 (31%)	
**DFS**	69 (45, 79)	76 (69, 81)	39 (28, 46)	<0.001

[1]Median (IQR); n (%)

[2]Wilcoxon rank sum test; Pearson's Chi-squared test; Fisher's exact test
