# Supplementary material for: FABP4/5-Mediated Lipid Reprogramming Is Associated with Immunosuppressive T Cell Crosstalk in Cervical Cancer After Chemoradiotherapy
Source: Biomedicines. 2026 Sep 15;14(9):2072. doi: 10.3390/biomedicines14092072 (PMC13604168; doi:10.3390/biomedicines14092072)
Supplement: Supplementary file 1 [file biomedicines-14-02072-s001.zip › Supplementary Table S3.pdf]

Table S3. Association between patient survival status and clinicopathological characteristics or immunohistochemical marker expression

Variable	Overall, N = 101[1]	Alive, N = 72[1]	Dead, N = 29[1]	p-value[2]
**FABP5**	30,806 (5,701, 52,355)	23,475 (3,794, 43,914)	54,674 (13,179, 112,672)	0.003
**FABP4**	1,994 (374, 10,323)	1,036 (308, 4,364)	13,502 (2,745, 32,165)	<0.001
**CD25**	60 (30, 102)	47 (26, 80)	139 (80, 167)	<0.001
**Age**	46 (41, 53)	44 (39, 47)	56 (48, 62)	<0.001
**T**				<0.001
T1	55 (54%)	51 (71%)	4 (14%)	
T2	28 (28%)	14 (19%)	14 (48%)	
T3	17 (17%)	7 (9.7%)	10 (34%)	
T4	1 (1.0%)	0 (0%)	1 (3.4%)	
**N**				<0.001
N0	84 (83%)	66 (92%)	18 (62%)	
N1	17 (17%)	6 (8.3%)	11 (38%)	
**M**				
M0	101 (100%)	72 (100%)	29 (100%)	
**DFS**	69 (45, 79)	75 (68, 81)	36 (28, 43)	<0.001
**Recurrence**	35 (35%)	6 (8.3%)	29 (100%)	<0.001
**Clinical_stage**				<0.001
1	55 (54%)	51 (71%)	4 (14%)	
2	28 (28%)	14 (19%)	14 (48%)	
3	18 (18%)	7 (9.7%)	11 (38%)	
**Histotype**				0.009
Ⅰ	16 (16%)	16 (22%)	0 (0%)	
Ⅰ-Ⅱ	5 (5.0%)	2 (2.8%)	3 (10%)	
Ⅱ	7 (6.9%)	6 (8.3%)	1 (3.4%)	
Ⅱ-Ⅲ	10 (9.9%)	6 (8.3%)	4 (14%)	

III	63 (62%)	42 (58%)	21 (72%)	
**Pathological_type**				0.019
AC	3 (3.0%)	2 (2.8%)	1 (3.4%)	
ASC	4 (4.0%)	3 (4.2%)	1 (3.4%)	
ASCUS	15 (15%)	15 (21%)	0 (0%)	
SCC	79 (78%)	52 (72%)	27 (93%)	

[1]Median (IQR); n (%)

[2]Wilcoxon rank sum test; Fisher's exact test; Pearson's Chi-squared test
