# Supplementary material for: FABP4/5-Mediated Lipid Reprogramming Is Associated with Immunosuppressive T Cell Crosstalk in Cervical Cancer After Chemoradiotherapy
Source: Biomedicines. 2026 Sep 15;14(9):2072. doi: 10.3390/biomedicines14092072 (PMC13604168; doi:10.3390/biomedicines14092072)
Supplement: Supplementary file 1 [file biomedicines-14-02072-s001.zip › Supplementary Table S4.pdf]

Table S4. Comparison of gene expression and clinical features among different pathological types

Variable	Overall, N = 101[1]	AC, N = 3[1]	ASC, N = 4[1]	ASCUS, N = 15[1]	SCC, N = 79[1]	p-value[2]
**FABP5**	30,806 (5,701, 52,355)	2,863 (1,478, 74,472)	19,559 (110, 46,886)	17,456 (8,783, 32,277)	31,821 (9,083, 59,125)	0.3
**FABP4**	1,994 (374, 10,323)	2,265 (1,684, 56,165)	296 (192, 2,578)	1,200 (433, 3,606)	2,599 (432, 13,818)	0.4
**CD25**	60 (30, 102)	102 (82, 130)	24 (18, 45)	35 (24, 69)	62 (34, 112)	0.053
**Survival_status (Dead)**	29 (29%)	1 (33%)	1 (25%)	0 (0%)	27 (34%)	0.019
**Age**	46 (41, 53)	48 (48, 58)	45 (42, 51)	46 (40, 54)	46 (41, 53)	0.5
**T**						0.006
T1	55 (54%)	1 (33%)	2 (50%)	15 (100%)	37 (47%)	
T2	28 (28%)	1 (33%)	1 (25%)	0 (0%)	26 (33%)	
T3	17 (17%)	1 (33%)	1 (25%)	0 (0%)	15 (19%)	
T4	1 (1.0%)	0 (0%)	0 (0%)	0 (0%)	1 (1.3%)	
**N**						0.12
N0	84 (83%)	2 (67%)	3 (75%)	15 (100%)	64 (81%)	
N1	17 (17%)	1 (33%)	1 (25%)	0 (0%)	15 (19%)	
**M**						
M0	101 (100%)	3 (100%)	4 (100%)	15 (100%)	79 (100%)	
**DFS**	69 (45, 79)	41 (35, 50)	66 (54, 68)	77 (69, 80)	68 (42, 78)	0.027
**Recurrence**	35 (35%)	3 (100%)	1 (25%)	1 (6.7%)	30 (38%)	0.005
**Clinical_stage**						0.002
1	55 (54%)	1 (33%)	2 (50%)	15 (100%)	37 (47%)	
2	28 (28%)	1 (33%)	1 (25%)	0 (0%)	26 (33%)	

3	18 (18%)	1 (33%)	1 (25%)	0 (0%)	16 (20%)	
**Histotype**						<0.001
I	16 (16%)	1 (33%)	0 (0%)	15 (100%)	0 (0%)	
I - II	5 (5.0%)	0 (0%)	0 (0%)	0 (0%)	5 (6.3%)	
II	7 (6.9%)	0 (0%)	0 (0%)	0 (0%)	7 (8.9%)	
II -III	10 (9.9%)	0 (0%)	0 (0%)	0 (0%)	10 (13%)	
III	63 (62%)	2 (67%)	4 (100%)	0 (0%)	57 (72%)	

[1]Median (IQR); n (%)

[2]Kruskal-Wallis rank sum test; Fisher's exact test
