# Supplementary material for: FABP4/5-Mediated Lipid Reprogramming Is Associated with Immunosuppressive T Cell Crosstalk in Cervical Cancer After Chemoradiotherapy
Source: Biomedicines. 2026 Sep 15;14(9):2072. doi: 10.3390/biomedicines14092072 (PMC13604168; doi:10.3390/biomedicines14092072)
Supplement: Supplementary file 1 [file biomedicines-14-02072-s001.zip › Supplementary Table S5.pdf]

Table S5: Comparison of gene expression and clinical features among different pathological types in the GSE52903 dataset.

Variable	Overall, N = 39[1]	ACC, N = 12[1]	ASCC, N = 1[1]	SCC, N = 26[1]	p-value[2]
**FABP4**	2.34 (2.25, 2.40)	2.28 (2.21, 2.33)	2.20 (2.20, 2.20)	2.36 (2.28, 2.52)	0.062
**FABP5**	3.28 (3.03, 3.67)	2.99 (2.89, 3.13)	2.94 (2.94, 2.94)	3.62 (3.27, 3.68)	<0.001
**Stage**					0.7
IB1	15 (38%)	6 (50%)	0 (0%)	9 (35%)	
IB2	14 (36%)	4 (33%)	0 (0%)	10 (38%)	
IIA	1 (2.6%)	0 (0%)	0 (0%)	1 (3.8%)	
IIB	7 (18%)	2 (17%)	1 (100%)	4 (15%)	
IIIB	2 (5.1%)	0 (0%)	0 (0%)	2 (7.7%)	
**Age**	42 (34, 54)	36 (34, 47)	55 (55, 55)	44 (34, 54)	0.3
**Treatment**					0.6
HT	5 (13%)	3 (25%)	0 (0%)	2 (7.7%)	
HT+TELE+BRACHY	5 (13%)	1 (8.3%)	0 (0%)	4 (15%)	
HT+TELE+BRACHY+CHEMO	1 (2.6%)	1 (8.3%)	0 (0%)	0 (0%)	
TELE+BRACHY	4 (10%)	1 (8.3%)	0 (0%)	3 (12%)	
TELE+BRACHY+CHEMO	20 (51%)	5 (42%)	1 (100%)	14 (54%)	
TELE+BRACHY+HT	1 (2.6%)	1 (8.3%)	0 (0%)	0 (0%)	
TELE+CHEMO+HT	2 (5.1%)	0 (0%)	0 (0%)	2 (7.7%)	

TELE+HT	1 (2.6%)	0 (0%)	0 (0%)	1 (3.8%)	
**Follow-up**	60 (38, 64)	58 (18, 62)	59 (59, 59)	61 (42, 66)	0.6
**Survival**					0.4
Alive	28 (72%)	8 (67%)	1 (100%)	19 (73%)	
Death	9 (23%)	2 (17%)	0 (0%)	7 (27%)	
Unknown	2 (5.1%)	2 (17%)	0 (0%)	0 (0%)	

[1]Median (IQR); n (%)

[2]Kruskal-Wallis rank sum test; Fisher's exact test
