# Supplementary material for: FABP4/5-Mediated Lipid Reprogramming Is Associated with Immunosuppressive T Cell Crosstalk in Cervical Cancer After Chemoradiotherapy
Source: Biomedicines. 2026 Sep 15;14(9):2072. doi: 10.3390/biomedicines14092072 (PMC13604168; doi:10.3390/biomedicines14092072)
Supplement: Supplementary file 1 [file biomedicines-14-02072-s001.zip › Supplementary Table S6.pdf]

Table S6. Molecular and Clinical Characteristics Across Different Clinical Stages

Variable	Overall, N = 101[1]	G1, N = 55[1]	G2, N = 28[1]	G3, N = 18[1]	p-value[2]
**FABP5**	30,806 (5,701, 52,355)	22,752 (1,782, 43,726)	30,238 (9,460, 74,204)	41,939 (17,586, 102,019)	0.043
**FABP4**	1,994 (374, 10,323)	797 (281, 2,662)	6,395 (377, 13,046)	19,084 (5,247, 53,034)	<0.001
**CD25**	60 (30, 102)	41 (26, 69)	90 (60, 131)	124 (49, 175)	<0.001
**Survival_status (Dead)**	29 (29%)	4 (7.3%)	14 (50%)	11 (61%)	<0.001
**Age**	46 (41, 53)	45 (40, 50)	50 (43, 62)	48 (44, 54)	0.035
**Pathological_type**					0.002
AC	3 (3.0%)	1 (1.8%)	1 (3.6%)	1 (5.6%)	
ASC	4 (4.0%)	2 (3.6%)	1 (3.6%)	1 (5.6%)	
ASCUS	15 (15%)	15 (27%)	0 (0%)	0 (0%)	
SCC	79 (78%)	37 (67%)	26 (93%)	16 (89%)	
**Histotype**					<0.001
I	16 (16%)	16 (29%)	0 (0%)	0 (0%)	
I - II	5 (5.0%)	2 (3.6%)	2 (7.1%)	1 (5.6%)	
II	7 (6.9%)	3 (5.5%)	4 (14%)	0 (0%)	
II -III	10 (9.9%)	6 (11%)	4 (14%)	0 (0%)	
III	63 (62%)	28 (51%)	18 (64%)	17 (94%)	
**T**					<0.001
T1	55 (54%)	55 (100%)	0 (0%)	0 (0%)	
T2	28 (28%)	0 (0%)	28 (100%)	0 (0%)	
T3	17 (17%)	0 (0%)	0 (0%)	17 (94%)	
T4	1 (1.0%)	0 (0%)	0 (0%)	1 (5.6%)	
**N**					<0.001
N0	84 (83%)	55 (100%)	28 (100%)	1 (5.6%)	

N1	17 (17%)	0 (0%)	0 (0%)	17 (94%)	
**M**					
M0	101 (100%)	55 (100%)	28 (100%)	18 (100%)	
**DFS**	69 (45, 79)	73 (68, 80)	46 (32, 77)	46 (30, 67)	<0.001
**Recurrence**	35 (35%)	7 (13%)	17 (61%)	11 (61%)	<0.001

[1]Median (IQR); n (%)

[2]Kruskal-Wallis rank sum test; Pearson's Chi-squared test; Fisher's exact test
