# Supplementary material for: FABP4/5-Mediated Lipid Reprogramming Is Associated with Immunosuppressive T Cell Crosstalk in Cervical Cancer After Chemoradiotherapy
Source: Biomedicines. 2026 Sep 15;14(9):2072. doi: 10.3390/biomedicines14092072 (PMC13604168; doi:10.3390/biomedicines14092072)
Supplement: Supplementary file 1 [file biomedicines-14-02072-s001.zip › Supplementary Table S7.pdf]

Table S7. Expression profiles of immunohistochemical markers in cervical cancer tissues across histological grades

Variable	Overall, N = 101[1]	Ⅰ, N = 16[1]	Ⅰ-Ⅱ, N = 5[1]	Ⅱ, N = 7[1]	Ⅱ-Ⅲ, N = 10[1]	Ⅲ, N = 63[1]	p-value[2]
**FABP5**	30,806 (5,701, 52,355)	14,988 (6,499, 31,997)	13,180 (13,179, 18,368)	17,883 (14,692, 46,508)	37,866 (18,845, 68,864)	38,153 (4,548, 65,970)	0.3
**FABP4**	1,994 (374, 10,323)	1,210 (480, 3,201)	2,119 (89, 2,745)	599 (258, 2,234)	623 (50, 13,628)	3,272 (562, 14,348)	0.2
**CD25**	60 (30, 102)	36 (26, 66)	77 (59, 88)	87 (44, 106)	95 (51, 121)	57 (30, 110)	0.3
**Survival_status**	29 (29%)	0 (0%)	3 (60%)	1 (14%)	4 (40%)	21 (33%)	0.009
**Overall_survival**	71 (52, 79)	77 (70, 80)	53 (48, 84)	81 (72, 82)	74 (53, 76)	68 (42, 76)	0.049
**Age**	46 (41, 53)	46 (40, 52)	53 (51, 60)	43 (37, 50)	43 (40, 47)	46 (42, 54)	0.2
**Pathological_type**							<0.001
AC	3 (3.0%)	1 (6.2%)	0 (0%)	0 (0%)	0 (0%)	2 (3.2%)	
ASC	4 (4.0%)	0 (0%)	0 (0%)	0 (0%)	0 (0%)	4 (6.3%)	
ASCUS	15 (15%)	15 (94%)	0 (0%)	0 (0%)	0 (0%)	0 (0%)	
SCC	79 (78%)	0 (0%)	5 (100%)	7 (100%)	10 (100%)	57 (90%)	
**T**							0.002
T1	55 (54%)	16 (100%)	2 (40%)	3 (43%)	6 (60%)	28 (44%)	
T2	28 (28%)	0 (0%)	2 (40%)	4 (57%)	4 (40%)	18 (29%)	
T3	17 (17%)	0 (0%)	1 (20%)	0 (0%)	0 (0%)	16 (25%)	
T4	1 (1.0%)	0 (0%)	0 (0%)	0 (0%)	0 (0%)	1 (1.6%)	

**N**							0.031
N0	84 (83%)	16 (100%)	4 (80%)	7 (100%)	10 (100%)	47 (75%)	
N1	17 (17%)	0 (0%)	1 (20%)	0 (0%)	0 (0%)	16 (25%)	
**M**							
M0	101 (100%)	16 (100%)	5 (100%)	7 (100%)	10 (100%)	63 (100%)	
**DFS**	69 (45, 79)	77 (68, 80)	48 (46, 84)	81 (72, 82)	74 (53, 76)	67 (40, 74)	0.054
**Recurrence**	35 (35%)	2 (12%)	3 (60%)	1 (14%)	4 (40%)	25 (40%)	0.12
**Clinical_stage**							<0.001
1	55 (54%)	16 (100%)	2 (40%)	3 (43%)	6 (60%)	28 (44%)	
2	28 (28%)	0 (0%)	2 (40%)	4 (57%)	4 (40%)	18 (29%)	
3	18 (18%)	0 (0%)	1 (20%)	0 (0%)	0 (0%)	17 (27%)	

[1]Median (IQR); n (%)

[2]Kruskal-Wallis rank sum test; Fisher's exact test
