# Supplementary material for: FABP4/5-Mediated Lipid Reprogramming Is Associated with Immunosuppressive T Cell Crosstalk in Cervical Cancer After Chemoradiotherapy
Source: Biomedicines. 2026 Sep 15;14(9):2072. doi: 10.3390/biomedicines14092072 (PMC13604168; doi:10.3390/biomedicines14092072)
Supplement: Supplementary file 1 [file biomedicines-14-02072-s001.zip › Supplementary Table S8.pdf]

Variable	Overall, N = 101[1]	FABP4_High, N = 51[1]	FABP4_Low, N = 50[1]	p-value[2]
**FABP5**	31,452 (8,588, 52,645)	45,750 (22,678, 90,383)	14,919 (1,203, 32,557)	<0.001
**FABP4**	1,994 (374, 10,323)	10,323 (4,380, 30,126)	370 (89, 853)	<0.001
**CD25**	60 (30, 102)	74 (40, 132)	52 (25, 92)	0.025
**Survival_status(Dead)**	29 (29%)	24 (47%)	5 (10%)	<0.001
**Age**	46 (41, 53)	47 (44, 56)	44 (39, 50)	0.008
**Pathological_type**				0.7
AC	3 (3.0%)	2 (3.9%)	1 (2.0%)	
ASC	4 (4.0%)	1 (2.0%)	3 (6.0%)	
ASCUS	15 (15%)	7 (14%)	8 (16%)	
SCC	79 (78%)	41 (80%)	38 (76%)	
**Histotype**				0.3
Ⅰ	16 (16%)	8 (16%)	8 (16%)	
Ⅰ-Ⅱ	5 (5.0%)	3 (5.9%)	2 (4.0%)	
Ⅱ	7 (6.9%)	1 (2.0%)	6 (12%)	
Ⅱ-Ⅲ	10 (9.9%)	4 (7.8%)	6 (12%)	
Ⅲ	63 (62%)	35 (69%)	28 (56%)	
**T**				<0.001
T1	55 (54%)	18 (35%)	37 (74%)	
T2	28 (28%)	17 (33%)	11 (22%)	
T3	17 (17%)	15 (29%)	2 (4.0%)	
T4	1 (1.0%)	1 (2.0%)	0 (0%)	
**N**				<0.001
N0	84 (83%)	36 (71%)	48 (96%)	
N1	17 (17%)	15 (29%)	2 (4.0%)	
**M**				

M0	101 (100%)	51 (100%)	50 (100%)	
**Clinical_stage**				<0.001
1	55 (54%)	18 (35%)	37 (74%)	
2	28 (28%)	17 (33%)	11 (22%)	
3	18 (18%)	16 (31%)	2 (4.0%)	
**Recurrence**	35 (35%)	25 (49%)	10 (20%)	0.002
**DFS**	69 (45, 79)	65 (38, 76)	72 (67, 81)	0.003

[1]Median (IQR); n (%)

[2]Wilcoxon rank sum test; Pearson's Chi-squared test; Fisher's exact test
