# Supplementary material for: FABP4/5-Mediated Lipid Reprogramming Is Associated with Immunosuppressive T Cell Crosstalk in Cervical Cancer After Chemoradiotherapy
Source: Biomedicines. 2026 Sep 15;14(9):2072. doi: 10.3390/biomedicines14092072 (PMC13604168; doi:10.3390/biomedicines14092072)
Supplement: Supplementary file 1 [file biomedicines-14-02072-s001.zip › Supplementary Table S9.pdf]

Variable	Overall, N = 101[1]	FABP5_High, N = 51[1]	FABP5_Low, N = 50[1]	p-value[2]
**FABP5**	31,452 (8,588, 52,645)	52,645 (41,390, 94,582)	7,120 (373, 15,789)	<0.001
**FABP4**	1,994 (374, 10,323)	8,145 (871, 28,824)	926 (105, 2,708)	<0.001
**CD25**	60 (30, 102)	66 (35, 126)	54 (28, 91)	0.2
**Survival_status(Dead)**	29 (29%)	18 (35%)	11 (22%)	0.14
**Age**	46 (41, 53)	47 (42, 56)	45 (39, 52)	0.12
**Pathological_type**				0.8
AC	3 (3.0%)	1 (2.0%)	2 (4.0%)	
ASC	4 (4.0%)	2 (3.9%)	2 (4.0%)	
ASCUS	15 (15%)	6 (12%)	9 (18%)	
SCC	79 (78%)	42 (82%)	37 (74%)	
**Histotype**				0.4
Ⅰ	16 (16%)	6 (12%)	10 (20%)	
Ⅰ-Ⅱ	5 (5.0%)	1 (2.0%)	4 (8.0%)	
Ⅱ	7 (6.9%)	3 (5.9%)	4 (8.0%)	
Ⅱ-Ⅲ	10 (9.9%)	5 (9.8%)	5 (10%)	
Ⅲ	63 (62%)	36 (71%)	27 (54%)	
**T**				0.062
T1	55 (54%)	24 (47%)	31 (62%)	
T2	28 (28%)	14 (27%)	14 (28%)	
T3	17 (17%)	13 (25%)	4 (8.0%)	
T4	1 (1.0%)	0 (0%)	1 (2.0%)	
**N**				0.069
N0	84 (83%)	39 (76%)	45 (90%)	
N1	17 (17%)	12 (24%)	5 (10%)	
**M**				

M0	101 (100%)	51 (100%)	50 (100%)	
**Clinical_stage**				0.11
1	55 (54%)	24 (47%)	31 (62%)	
2	28 (28%)	14 (27%)	14 (28%)	
3	18 (18%)	13 (25%)	5 (10%)	
**Recurrence**	35 (35%)	21 (41%)	14 (28%)	0.2
**DFS**	69 (45, 79)	68 (40, 77)	70 (54, 80)	0.3

[1]Median (IQR); n (%)

[2]Wilcoxon rank sum test; Pearson's Chi-squared test; Fisher's exact test
